# Genes Participating in the Ensheathment of Neurons Are Affected by Postnatal Stress and Maternal Immune Activation in the Pituitary Gland

**DOI:** 10.3390/genes14051007

**Published:** 2023-04-28

**Authors:** Samah Alsegehy, Bruce R. Southey, Laurie Rund, Rodney W. Johnson, Sandra L. Rodriguez-Zas

**Affiliations:** 1 School of Information Sciences, University of Illinois at Urbana-Champaign, Urbana, IL 61820, USA; 2 Department of Animal Sciences, University of Illinois at Urbana-Champaign, Urbana, IL 61801, USA; 3 Division of Nutritional Sciences, University of Illinois at Urbana-Champaign, Urbana, IL 61801, USA; 4 Neuroscience Program, University of Illinois at Urbana-Champaign, Urbana, IL 61801, USA; 5 Department of Statistics, University of Illinois at Urbana-Champaign, Urbana, IL 61820, USA

**Keywords:** RNA-seq, pituitary gland, ensheathment of neurons

## Abstract

Immune challenges during gestation are associated with neurodevelopmental disorders and can interact with stress later in life. The pituitary gland participates in endocrine- and immune-related processes that influence development, growth, and reproduction and can modulate physiological and behavioral responses to challenges. The objective of this study was to investigate the effect of stressors at different time points on the molecular mechanisms of the pituitary gland and detect sex differences. RNA sequencing was used to profile the pituitary glands of female and male pigs exposed to weaning stress and virally induced maternal immune activation (MIA), relative to unchallenged groups. Significant effects (FDR-adjusted *p*-value < 0.05) of MIA and weaning stress were detected in 1829 and 1014 genes, respectively. Of these, 1090 genes presented significant interactions between stressors and sex. The gene ontology biological process of the ensheathment of neurons (GO:0007272), substance abuse, and immuno-related pathways, including the measles disease (ssc05162), encompasses many genes with profiles impacted by MIA and weaning stress. A gene network analysis highlighted the under-expression of myelin protein zero (*Mpz*) and inhibitors of DNA binding 4 (*Id4*) among the non-stressed males exposed to MIA, relative to the control and non-MIA males exposed to weaning stress, relative to non-stressed pigs. The detection of changes in the molecular mechanisms of the pituitary gland could advance our understanding of disruptions in the formation of the myelin sheath and the transmission of neuron-to-neuron signals in behavioral disorders associated with maternal immune activation and stress.

## 1. Introduction

Immune challenges during gestation can influence the likelihood of neurodevelopmental disorders and a susceptibility to stressors later in life. During gestation, mothers can be exposed to various challenges, resulting in an immune response known as maternal immune activation (MIA) [1]. The activation of immune signaling is associated with increased levels of glucocorticoid and corticosterone in offspring [2]. This immune signaling can also disturb the central nervous system’s development and is a contributing risk factor for psychiatric afflictions such as major depressive disorder (MDD), schizophrenia spectrum disorder (SSD), and autism spectrum disorder (ASD) [3].

Stress is the biological response to an event perceived as threatening homeostasis, and weaning is an example of such stress [4]. Depending on the circumstance and species, weaning can include stress due to the removal of offspring from their mother and siblings’ side, handling and transportation, new housing, and diet [5,6]. Weaning alters the immune homeostasis of the offspring due to a transition from the passive immunity of mother’s milk to a development of the offspring’s active immunity [6]. Weaning stress activates the hypothalamus–pituitary–adrenal (HPA) axis, releasing stress hormones and inflammation in the body [7]. The pituitary gland is central in regulating the physiological and behavioral stress response [8,9]. The pituitary gland affects endocrine function and regulates development, growth, and reproduction by mediating the transmission of hypothalamic signals to target organs. Aberrations in the HPA axis, including those specific to the pituitary gland, have been associated with MIA, with alterations in its structure and the neural circuitries linked to depression [10,11].

Maternal immune activation is associated with changes in the expression of genes in the brain, including adenylate cyclase-activating polypeptide 1 (*Adcyap1*), a gene that codes pituitary adenylate cyclase-activating peptide (PACAP) neuropeptides [12]. In the pituitary gland, the products of PACAP stimulate the formation of cyclic adenosine monophosphate (CAMP). This messenger transduces the signals of hormones into cells and regulates the secretion of the hormones responsible for vasodilation and immune suppression. Similarly, in response to stress, such as that induced by weaning, the pituitary gland releases vasopressin (AVP) products and the corticotrophin-releasing factor modulates corticosteroids secretion via the adrenal gland [13]. The mRNAs of the receptors of the cytokine interleukins 17 IL-17rc and IL-17rd are abundant in the pituitary, making this gland susceptible to cytokine signaling [14]. Moreover, the physiological and behavioral response to postnatal stress can be affected by MIA [15]. While the effects of MIA and its stress on specific molecules in the pituitary gland have been reported, there is limited information on the simultaneous effect of MIA and weaning on the pituitary gland’s transcriptome. This study aimed to comprehensively characterize the impacts of immune activation during gestation and the postnatal stress of weaning on the pituitary gene networks. The pituitary gland influences the development and growth of sex organs and reproduction; therefore, the effects of stressors before and after birth were studied in females and males. The pituitary transcriptome was profiled in the established model of pigs prenatally exposed to viral immune activation and weaning stress 60 days after inoculation.

## 2. Materials and Methods

The animal studies were approved by the Institutional Animal Care and Use Committee (IACUC) at the University of Illinois and the protocols have been described [16]. Succinctly, the gestating gilts (Camborough breed) were moved into individual disease containment chambers at gestation day 69. The gilts were housed in a 12 h light/dark cycle, receiving water ad libitum and feed to match their nutritional needs at each physiological stage of the experiment [17]. Among the 12 gilts, half were challenged on gestation day 76 with the porcine respiratory and reproductive virus (PRRSV). The challenged gilts received an intranasal inoculation of strain P129-BV at a dose of 5 mL of 1 × 10^5^ median tissue culture mixed with Dulbecco’s modified Eagle medium [1]. The control gilts were inoculated with 5 mL of the medium. ELISA and PCR testing verified the infection status of the gilts, which was confirmed by the decreased food intake and increased body temperatures of the infected females, which extended two weeks after inoculation [17].

After farrowing at gestation day 113, the offspring remained with their mothers until 21 days of age, when half of the pigs were removed from the litter. The weaned pigs were housed in groups of 4 to 5 pigs per chamber and received water ad libitum and a diet adequate for the growing stage of life. On day 22, all the pigs were anesthetized with a drug cocktail, including telazol, ketamine, and xylazine (Fort Dodge Animal Health, Fort Dodge, IA, USA) [17]. An intracardial injection of sodium pentobarbital (Vortech Pharmaceuticals, Dearborn, MI, USA) was used to euthanize the pigs. The pituitary glands were extracted, flash-frozen, and maintained at −80 °C [17]. The EZNA isolation kit (Omega Biotek, Norcross, GA, USA) was used to isolate the RNA [17]. Low RNA degradation was ensured by sequencing the samples with an RNA integrity number higher than 7.2. Altogether, the pituitary glands from 48 animals were analyzed, including 24 samples per prenatal treatment group (MIA or control), 24 samples per postnatal stress group (weaning or nursed), and 24 samples per sex group. The distribution of these samples was balanced across the groups and a depiction of the experimental design is available in Supplemental Figure S1.

### 2.1. Pituitary Gland RNA Sequencing and Analysis

The RNAseq libraries were prepared using the TruSeq Stranded mRNAseq Sample Prep kit (Illumina Inc, San Diego, CA, USA) [17]. The libraries were sequenced on NovaSeq 6000 equipment, using the S4 reagent kit for approximately 150 cycles from each end to generate 150 bp paired-end reads [7]. The software bcl2fastq v2.20 (Illumina Inc, San Diego, CA, USA) was used to demultiplex and produce FASTQ files. Quality control of all the samples using the software FASTQC [18] indicated that all the read positions had a minimum Phred score of 30; therefore, no reads were trimmed. The paired-end reads were aligned to the Sus scrofa transcriptome version Sscrofa v. 11.1 [19] and quantified using kallisto v. 0.43.0 [20] with its default settings. The read count per gene was normalized for the library size and transcript length, and gene profiles supported by more than five transcripts per million RNA molecules [21] for each weaning–MIA–sex combination were analyzed using the edgeR v. 3.22.5 software in the environment R v. 3.5 [22]. The effects of weaning stress, MIA, sex, and two-way interactions were tested. For the main effects, positive log_2_(fold change) values indicated higher levels of expression in the first group (control, stressed, or males) compared to the second group (MIA, nursed, or females), and the reverse profile corresponded to a negative log_2_(fold change). For the interactions, positive log_2_(fold change) values indicated over-expression in the groups exposed to one challenge (MIA or stress) or the males exposed to either challenge, relative to the other groups. In contrast, negative log_2_(fold change) values indicated over-expression in the groups exposed to both challenges (MIA and stress) or the females exposed to either challenge, relative to the other groups. The group comparison order was stressed (weaned) compared to unstressed (nursed) pigs, non-virally challenged (control) compared to MIA-exposed pigs, and males compared to females. The RNA sequences are available in the Gene Expression Omnibus database, study GSE224944 [23].

### 2.2. Functional Enrichment, Network Inference, and Transcriptional Factor Analysis

Gene Set Enrichment Analysis (GSEA) and Over-Representation Analysis (ORA) approaches were used to identify the functional categories potentially impacted by weaning stress, MIA, and sex effects. The over-representation of Kyoto Encyclopedia of Genes and Genomes (KEGG) pathways and the Gene Ontology (GO) Biological Process (BP) [24,25,26] was explored. The WebGESTALT software with its default settings and the Sus scrofa genome as a reference were used to estimate the functional category enrichment score [27].

The ORA approach identified the categories among the significantly differentially expressed genes (FDR-adjusted *p*-value < 0.05), irrespective of profile. The enrichment ratio indicates the prevalence of differentially expressed genes in a category, relative to all the genes and all the categories. The GSEA approach incorporates the ranked gene log_2_(fold change) and provides a normalized enrichment score (NES) for each category. The enrichment sign was calculated from the maximum deviation of the cumulative sum of the ranked genes divided by the average of the permutated enrichment scores, and the FDR-adjusted *p*-value was computed using 1000 permutations.

For the potential changes in the relationships between the genes caused by MIA and weaning, gene-network-based gene expression profiles were constructed with Cytoscape v. 3.9.1 [28] and the databases of molecular interactions based on protein, co-expression, text mining, and experimental information are available in the STRING repository [29]. The Sus scrofa database was used, and in the absence of a gene, the human database was considered. The network of the genes in the highly enriched categories was studied. The color of the gene node reflects the sign of the fold change, with red denoting under-expression and green denoting over-expression in the first group relative to the second group.

## 3. Results

### 3.1. Sequencing Differential Gene Expression Descriptive

The sequencing of the 48 samples produced approximately 6,253,000,000 reads. On average, 97,700,000 reads were obtained per sample, and this count was consistent across the treatment groups (7.3% maximum read count difference between groups). After filtering the genes for low read counts within the groups, the analysis was conducted on 16,585 genes. At the threshold of |log2(fold change between groups)| > 1.25 and an FDR-adjusted *p*-value of < 0.05, 456 genes had a significant MIA-by-weaning effect, 200 genes had a significant weaning-by-sex effect, 890 genes had a significant MIA-by-sex effect, 483 genes had a significant MIA effect, 358 genes had a significant weaning effect, and 821 genes had a significant sex effect. Supplemental Table S1 presents the differentially expressed genes (FDR-adjusted *p*-value < 0.05 and the |log_2_(fold change)| > 2.5) for all the effects analyzed.

### 3.2. Effects of Maternal Immune Activation, Stress, and Sex on the Pituitary Biological Processes and Genes

The GO biological processes and KEGG pathways that were enriched in the GSEA (FDR-adjusted *p*-value < 0.05 and NES > |1.6|) for at least two effects are presented in Table 1. An extended list of the enriched functional categories in the GSEA and ORA is available in Supplemental Table S2. Overall, the GSEA and ORA detected the enrichment of biological processes associated with nervous system development (e.g., the ensheathment of neurons GO:0007272, myelination GO:0042552, reproductive system development GO:0061458, gland development GO:0048732, and steroid biosynthetic process GO:0006694) and cell cycle (e.g., chromosome segregation GO:0007059). Consistent with these findings, the GSEA of the KEGG pathways (and related biological processes identified by the ORA) detected the enrichment of the nervous system, substance dependence (e.g., GABAergic synapse ssc04727, nicotine addiction ssc05033, and the modulation of chemical synaptic transmission GO:0050804), and cell cycle (e.g., cell cycle ssc04110). Notably, the GSEA of these biological processes enabled the detection of signal transduction enrichment (e.g., neuropeptide signaling pathway GO:0007218, second-messenger-mediated signaling GO:0019932, and G protein-coupled receptor signaling pathway GO:0007187). In addition, the GSEA and ORA studies of these pathways and processes enabled the detection of immune system enrichment (e.g., Th17 cell differentiation ssc04659, Th1 and Th2 cell differentiation ssc04658, cellular response to interferon-γ GO:0071346, and leukocyte chemotaxis GO:0030595). The GSEA enrichment of these nervous- and immune system-related categories for the sex effect was primarily detected in the interactions with weaning stress and MIA (Table 1). The ORA enrichment across all the gene profiles enabled the detection of the enrichment of nervous-system-related biological processes for the main effect of sex.

The sign of the NES informs about the prevalent differential expression pattern elicited by MIA, weaning stress, or sex within the categories (Table 1). The positive NES sign for the ensheathment of neurons (GO:0007272) indicates an under-expression of the genes in MIA relative to the control pigs and an under-expression in males relative to females. The negative NES sign for the immune system pathway indicates that the genes were over-expressed in MIA relative to the control pigs. In contrast, the positive NES sign for the nervous system and organ development processes informs that the genes were under-expressed in MIA relative to the control pigs (Supplemental Table S2). The negative sign of NES for the nervous system and substance dependence processes and pathways indicates that the genes were over-expressed in females relative to males.

The expression patterns of the differentially expressed genes in the enriched functional categories of the ensheathment of neurons and immune response are summarized in Table 2. The genes listed in Table 2 representing the ensheathment of neurons category include claudin 11 (*Cldn11*), oligodendrocyte transcription factor 2 (*Olig2*), mal, T cell differentiation protein (*Mal*), and proteolipid protein 1 (*Plp1*). The genes representing the immune response categories include C-X-C motif chemokine ligand 10 (*Cxcl0)*, C-X-C motif chemokine ligand 9 (*Cxcl9*), TNF receptor superfamily member 8 (*Tnfrsf8*), and growth differentiation factor 1 (*Gdf1*). Complementing the information in Table 2, a more extensive list of these differentially expressed genes (FDR-adjusted *p*-value < 0.05 and |log_2_(fold change between groups)| > 2.5) across the functional categories is available in Supplemental Table S1.

The profiles of the genes in Table 2 are aligned with the extended list of the differentially expressed genes in Supplemental Table S1. Most of the genes across the pathways and processes are over-expressed in the control relative to the MIA-exposed pigs, under-expressed in the weaned relative to the non-stressed pigs, and under-expressed in males relative to females. Likewise, most of the differentially expressed genes across the pathways and processes are over-expressed in the MIA-exposed, weaned-stressed pigs, in weaned males, and in MIA-exposed females relative to the other groups studied.

### 3.3. Networks of Genes in the Ensheathment of Neurons Process Affected by Maternal Immune Activation, Stress, and Sex in the Pituitary Gland

**Figure 1:**
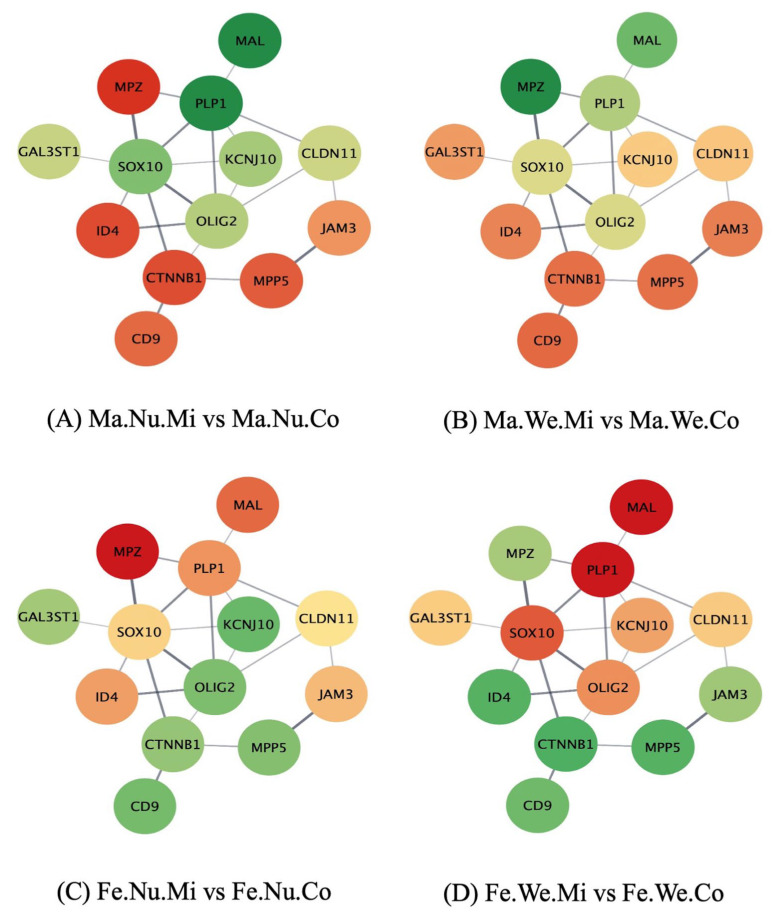
Comparison of maternal immune activation (Mi) versus (vs.) control (Co) within weaned (We), nursed (Nu), male (Ma), and female (Fe) groups in the network of genes (nodes) in the ensheathment of neurons biological process, where edges represent STRING database connections. Node color scheme: red to green denotes over- to under-expression in the first relative to the second group compared, respectively.

**Figure 2:**
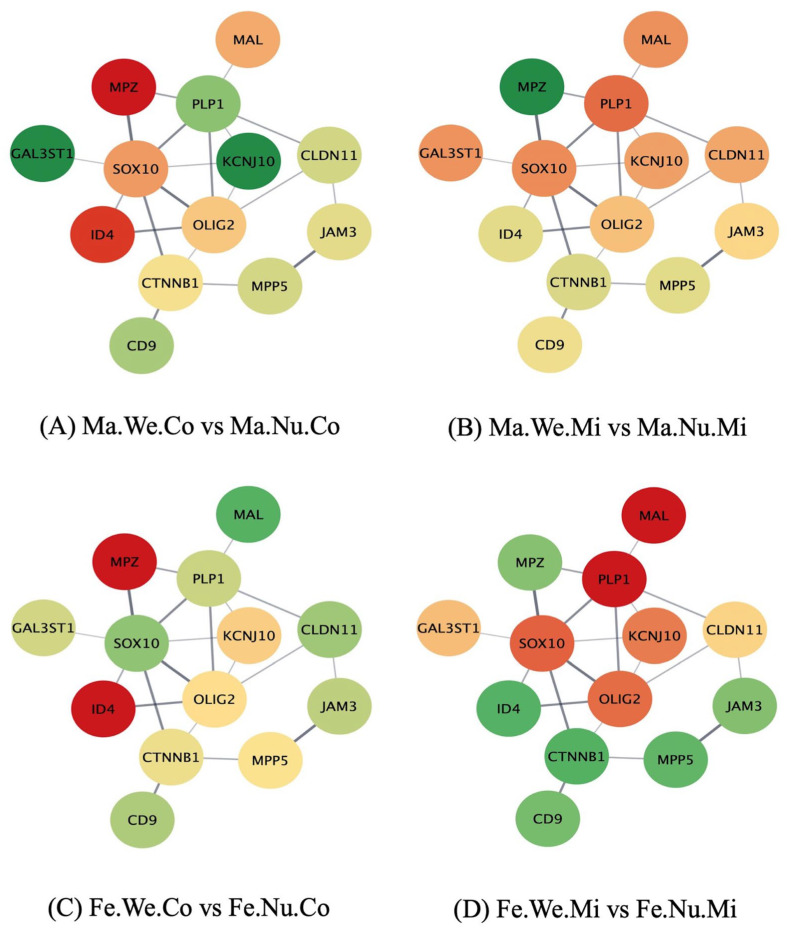
Comparison of weaned (We) versus (vs.) nursed (Nu) within maternal immune activation (Mi), control (Co), male (Ma), and female (Fe) groups in the network of genes (nodes) in the ensheathment of neurons biological process, where edges represent STRING database connections. Node color scheme: red to green denotes over- to under-expression in the first relative to the second group, respectively.

**Figure 3:**
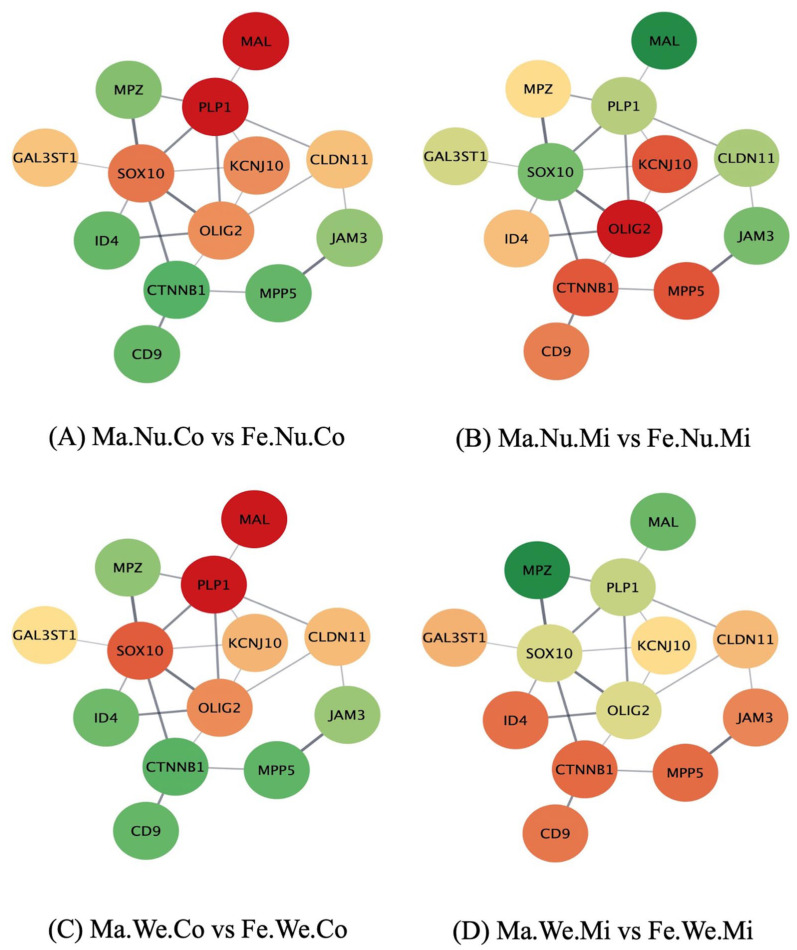
Comparison of male (Ma) versus (vs.) female (Fe) within maternal immune activation (Mi), control (Co), weaned (We), and nursed (Nu) groups in the network of genes (nodes) in the ensheathment of neurons biological process, where edges represent STRING database connections. Node color scheme: red to green denotes over- to under-expression in the first relative to the second group, respectively.

The study of the networks of the genes in the enriched pathways, using the molecular interaction information from the STRING database, furthered the comprehension of the effect of the factors studied on the relations between the gene profiles. Figure 1, Figure 2 and Figure 3 depict the networks of the genes in the ensheathment of neurons process, including 13 gene nodes in the human database and the expression profile in the pituitary gland between the groups that differ in MIA, weaning stress, or sex. The genes include myelin protein zero (*Mpz*), galactose-3-O-sulfotransferase 1 (*Gal3st1*), *Mal*, *Plp1*, SRY-box transcription factor 10 (*Sox10*), inhibitor of DNA binding 4 (*Id4*), potassium inwardly rectifying channel subfamily J member 10 (*Kcnj10*), *Olig2*, catenin β 1 (*Ctnnb1*), CD9 molecule (*Cd9*), *Cldn11*, junctional adhesion molecule 3 (*Jam3*), and membrane palmitoylated protein 5 (*Mpp5*). The color scheme of the nodes ranges from red to green, denoting over- and under-expression in the first relative to the second group, respectively. The log_2_(fold change) for each comparison are listed in Supplemental Table S3. The effect of MIA varied across the genes in the ensheathment of neurons network and depended on the second stress and sex of the individual.

The impact of MIA is characterized by the over-expression of *Mal* and *Plp1* and under-expression of *Mpz*, *Id4*, and *Ctnnb1* among the nursed MIA males compared to the nursed control males (Figure 1A). *Mpz* was over-expressed in the male weaned MIA group compared to the male weaned control pigs (Figure 1B), while *Mpz* was over-expressed in the nursed control females relative to the nursed MIA females (Figure 1C). Lastly, *Mal* and *Plp1* were under-expressed in the female weaned MIA group relative to the female weaned control pigs (Figure 1D).

The effect of weaning stress was evidenced in the over-expression of *Gal3st1* and *Kcnj10* and under-expression of *Mpz* and *Id4* in the male weaned control pigs compared to the male nursed control pigs (Figure 2A,B). On the other hand, *Mpz* and *Id4* were under-expressed in the female weaned control pigs compared to the female nursed control pigs, while most of the genes presented the opposite pattern (Figure 2C). Additionally, *Mpz* and *Id4* were over-expressed, whereas *Mal* and *Plp1* were under-expressed in the female weaned MIA group compared to the female nursed MIA pigs (Figure 2D).

The effect of sex included the under-expression of *Mal* and *Plp1* in males compared to females among the control pigs, irrespective of the second stress condition (Figure 3A,C). *Mal* was over-expressed and *Olig2* was under-expressed in males relative to females among the nursed pigs exposed to MIA (Figure 3B). Among the weaned MIA pigs, *Mpz* was over-expressed in males compared to females (Figure 3D).

## 4. Discussion

Prenatal immune challenges can impact offspring’s neurological processes and molecules during development and have a prolonged effect on their physiology, health, and behavior. The interaction between a prenatal challenge and a second challenge, such as stressful conditions after birth, is known as the double hit hypothesis and can result in either the sensitization or desensitization of the offspring’s neurological and molecular networks [30]. The present study pioneers an investigation of how MIA and second stress can impact the pituitary gland and complements studies on the effects of MIA and stressors on behavior, peripheral markers, the amygdala, and the hippocampus [1,12,16,31].

The enrichment of the processes and pathways underlying the nervous and immune systems (Table 1, Supplemental Table S2) is supported by the significant |log_2_(fold change between groups)| > 1.25, (FDR-adjusted *p*-value < 0.05) differential expression of 3208 genes in the pituitary gland, which were impacted by weaning stress, MIA, and sex effects acting independently or through interactions. Some examples of the GO biological processes and KEGG pathways enriched by these differentially expressed genes include neural ensheathment (GO:0007272), nicotine addiction (ssc05033), reproductive system development (GO:0061458), measles (ssc05162), and oxidative phosphorylation (ssc00190).

The enrichment of the biological processes of the ensheathment of neurons and myelination indicates changes in the genes associated with the formation of myelin sheath that are listed in Table 2. The myelin structure acts as a protective layer around the neurons, interfering in the transmission of the signals between neurons, and myelination abnormalities have been reported in MIA-related disorders such as SSD [32]. The positive NES for MIA and negative NES for the interaction of weaning-by-MIA indicates that the genes in this process are under-expressed in the pituitary gland of the pigs exposed to MIA and to weaning stress. This pattern suggests a synergistic effect of both stressors. A positive NES for MIA is also observed in the GO neurodevelopmental processes of the morphogenesis of a branching structure (GO:0001763) and pattern specification (GO:0007389). The observed profiles can be associated with reports that unmyelinated fibers in autonomic nerves co-regulate hormone secretion together with the endocrine fibers of the pituitary gland [33].

The under-expression of genes in the ensheathment of neurons and myelination processes advances our understanding of the effect of MIA, because hypomyelination has been associated with pituitary hypogonadotropic hypogonadism, therefore impacting reproduction processes [34]. The under-expression of genes in the ensheathment of neurons process associated with MIA or weaning stress is consistent with reports that Poly(I:C)-elicited MIA in mice reduces myelination and axonal development in the hippocampus of offspring prior to reaching adulthood [32]. Alterations in myelination were also associated with hippocampal transcriptomic changes following MIA in nonhuman primates [35]. The reduced and delayed myelination and associated neural abnormalities caused by MIA could be related to SSD-like behaviors.

The enrichment of the ensheathment of neurons process among the genes presenting MIA-by-sex is characterized by a negative NES for the sex effect, indicative of under-expression in males relative to females. This finding is consistent with reports that stress elicited by deprivation of mother–pup contact via early weaning reduces the formation of myelin in the brains of males but not female mice [36]. Additionally, consistent with the present findings, hypomyelination and social behavior deficits were observed in the male rats exposed to MIA [37].

The differentially expressed genes annotated to the ensheathment of neurons processes that presented significant differential expression included *Cldn11*, *Olig2*, *Mal*, *Plp1*, and *Mpz* (Table 2). These genes presented consistent patterns relative to other genes, including under-expression in MIA and over-expression in the nursed control females and control males, relative to other groups. Notably, this pattern highlights an antagonistic relationship between both stressors in the expression of genes in the ensheathment of neurons process. Consistent with our findings, *Cldn11* and *Mal* were under-expressed in the prefrontal cortex and nucleus accumbens of mice exposed to poly(I:C)-elicited MIA during gestation [38]. Cumulative prenatal and adult stress was associated with the under-expression of *Cldn11* in the motor cortex of rats [39]. Chronic inflammatory demyelinating polyradiculoneuropathy-like characteristics were associated with a mutation in *Mpz* that causes demyelination [40]. The under-expression of *Olig2* and *Plp1* was reported in the hippocampus of mice exposed to lipopolysaccharide-elicited MIA [41].

The enrichments of the reproductive system’s development and neuropeptide signaling pathways (GO:0007218) share a profile distinct from the ensheathment of neurons process. A positive NES characterizes the reproductive and prohormone pattern for MIA and the interaction of weaning-by-MIA, indicating that the genes in these processes are under-expressed in the pituitary gland of the pigs exposed to MIA, while over-expressed in the pigs exposed to weaning stress. This pattern suggests an antagonistic interaction between both stressors. Our findings are related to reports that exposure to toxic agents during development is a risk factor for male reproductive system development and sex hormone levels in the cord blood [42]. Related to our findings on hormonal signaling, studies have demonstrated that psychological and physical stresses, including infections, can impact the release of corticotropin-releasing hormone (CRH) from the hypothalamus [13]. The previous signals enhance the release of adrenocorticotropic hormone (ACTH) from the pituitary gland [43]. Additionally, *Mal*, *Plp1*, and *Olig2* were under-expressed in the prefrontal cortexes of mice susceptible to social defeat stress [44].

The enrichment of the nicotine and morphine addiction pathways (ssc05033 and ssc05032, respectively) shared patterns with the enrichment of the neuropeptide signaling pathway. These pathways include genes that participate in GABAergic and glutamatergic synapses and the balance of this transmission, which plays an important role in the response to prenatal stress and incidence of anxiety-like behaviors [45]. The previous category over-representation was characterized by a positive NES for the effect of the interaction of weaning-by-sex and a negative NES for the effect of weaning stress. This pattern corresponds to the pathway genes over-expressed in the nursed females, relative to the males exposed to weaning stress. This result is consistent with the over-expression of the genes annotated to the cocaine addiction pathway in the MIA-exposed nursed females, relative to weaned males, in the hippocampus [46].

The differentially expressed genes annotated to the nicotine addiction pathway (Table 1) included γ-aminobutyric acid type A receptor subunit alpha2 (*Gabra2*), γ-aminobutyric acid type A receptor subunit alpha3 (*Gabra3*), γ-aminobutyric acid type A receptor subunit alpha4 (*Gabra4*), glutamate ionotropic receptor NMDA type subunit 1 (*Grin1*), and glutamate ionotropic receptor NMDA type subunit 2A (*Grin2a*). These genes presented consistent patterns, including under-expression in weaned males relative to the other groups. Aligned with the present studies, *Gabra2* and *Gabra3* were under-expressed in various brain regions, including the hippocampus and amygdala, and in rats exposed to the ultrasound stress model [15,16,47]. Additionally, mutant mice lacking *Grin2a* expression do not exhibit anxiety-related behavior after restraint stress [48].

The enrichment of the immune system’s pathways is characterized by negative NES for MIA and positive NES for the interaction of weaning-by-MIA. The profiles of the genes in these pathways are summarized in Table 2. These patterns indicate that the genes in these pathways are over-expressed in the pituitary gland of the pigs exposed to MIA, while under-expressed in the pigs exposed to weaning stress. The gene expression profiles suggest an antagonistic interaction between the stressors, opposite to that observed in the reproductive system’s processes. The profiles observed in the present study were consistent with the over-expression of prefrontal cortex genes in the chemokine signaling pathway of the pigs exposed to MIA [49], and the under-expression of the genes annotated to the inflammation-associated pathways in the amygdala and hippocampus of the weaned pigs [16,46]. The importance of changes in the immune system pathway of the pituitary gland, in response to prenatal and weaning challenges, can also be related to the established immuno-enhancing role of many hormones secreted by the pituitary gland (e.g., prolactin, growth hormone, insulin growth factor-1, and thyroid hormones) in susceptibility to stress-induced disease [50].

The differentially expressed genes annotated to the immune response pathways included *Cxcl9*, *Cxcl10*, *Tnfrsf8*, and *Gdf1*. These genes presented consistent patterns, including over-expression in the not-stressed (nursed) MIA and female MIA pigs relative to the other groups. Notably, this pattern highlights an antagonistic relationship between the stressors in the over-expression of genes in the immune response pathways. In agreement with our findings, *Cxcl10* and *Tnfrsf8* were over-expressed in the hippocampus of the mice exposed to Poly(I:C)-elicited MIA, relative to the control mice [51].

Insights into the impact of prenatal and postnatal stressors were gained by considering the profile networks for the genes in the ensheathment of neurons process. The network connections and profiles of *Mpz*, *Id4*, *Mal*, and *Plp1* offered information on the effects of both stressors. In contrast, changes in *Ctnnb1* were associated with prenatal stress and changes in *Gal3st1* and *Kcnj10* were associated with postnatal stress. Notably, the submodule genes *Mpz* and *Id4* were under-expressed, whereas the submodule genes *Mal* and *Plp1* were over-expressed in the nursed MIA group, relative to the control males. The submodule genes *Mpz* and *Id4* were under-expressed in the weaned relative to nursed control males. The submodule genes *Mal* and *Plp1* were also over-expressed in the female weaned control and nursed groups, relative to the weaned MIA pigs. While *Mal* and *Plp1* were under-expressed, *Mpz* and *Id4* were over-expressed in males compared to females. The connection between stress and *Plp1* was confirmed in a study of the canine pituitary cortex that reported the differential expression of *Plp1* in association with stress tolerance [52].

Consistent with the profiles in the networks, the expression level of *Id4* decreased in the intermediate lobe of the pituitary glands of rats exposed to continuous stress conditions, which were modeled by high water levels in their cages [53]. *Gal3st1* and *Kcnj10* were over-expressed in the weaned relative to the nursed control males, whereas these genes were under-expressed in the weaned relative to the nursed MIA males. The impact of MIA on the profile of *Gal3st1* in response to stress may be related to reports that the expression of this gene decreases in the prefrontal cortex of individuals diagnosed with MIA-related SSD [54]. Likewise, the differential expression of *Kcnj10* was reported in the superior temporal cortex of individuals diagnosed with SSD [55].

## 5. Conclusions

This study advances our understanding of the effects of stress, such as weaning, following exposure to inflammatory signals during gestation on the pituitary gland transcriptome, which can result in neurodevelopmental disorders. This endocrine gland plays an important role in multiple functions because of the secretion of multiple endocrine hormones that target different cell types. The findings from our study offer evidence that the transcriptomic profile of the pituitary gland in a biomedical pig model is modulated by MIA during development and stresses such as weaning, and these effects can depend on the sex of the individual. The ensheathment of neurons and myelination functional categories were detected among the biological processes and pathways encompassing the genes impacted. Most genes in the previous processes (e.g., *Mpz*, *Mla*, *Id4*, and *Plp1*) presented MIA- or sex-dependent effects associated with the postnatal stressor. These results confirm the relevance of the double hit hypothesis in the endocrine gland transcriptome and the importance of studies that characterize the pituitary gland’s response to challenges in both sexes. The under-expression of genes in the myelination networks associated with MIA and stress detected in the present study may be associated with reports that unmyelinated fibers in the autonomic nerves interact with endocrine fibers to regulate hormone secretion and its related processes, such as circadian rhythms.

The effect of weaning stress on the expression of genes that participate in GABAergic and glutamatergic transmission was associated with the enrichment of addiction pathways. The balance of the previous signals can modulate the response to stressors, including behavioral disorders. The genes in these pathways were over-expressed in the nursed females relative to the other groups. The differential expression of genes in immune-related pathways, such as measles and primary immunodeficiency, was characterized by over-expression in the MIA females relative to the other groups. The implication of the effects of MIA and stress on the immune-related pathways is also relevant to the established role of many hormones secreted by the pituitary gland in immune system homeostasis. Our results encourage the study of the individual responses of the pituitary gland to challenges, in addition to a global analysis of the HPA axis, to further the understanding of the effect of developmental and postnatal challenges on physiology, health, and behavior.

## Figures and Tables

**Table 1:** Enriched pathways (Gene Set Enrichment Analysis False Discovery Rate adjusted FDR *p*-value < 0.05 and normalized enrichment score absolute value |NES| > 1.6) among genes presenting maternal immune activation (MIA), weaning, and sex, including interaction (×) effects in the pituitary gland.

Category	Identifier	MIA		Weaning		Sex		MIA × Sex		Weaning × Sex		Weaning × MIA	
		NES ^3^	FDR	NES	FDR	NES	FDR	NES	FDR	NES	FDR	NES	FDR
**GO Biological Process ^1^**													
Nervous system													
Ensheathment of neurons	GO:0007272	1.6	4 × 10^−2^			−1.8	2 × 10^−8^	1.8	5 × 10^−3^			−2.2	2 × 10^−8^
Regeneration	GO:0031099	1.6	4 × 10^−2^			−1.7	1 × 10^−2^	1.7	3 × 10^−2^				
Neuropeptide signaling pathway	GO:0007218	1.8	2 × 10^−2^	−1.9	3 × 10^−3^					1.9	5 × 10^−3^	1.8	2 × 10^−2^
Extracellular structure organization	GO:0043062	2.0	2 × 10^−8^					1.7	3 × 10^−2^				
Reproductive system development	GO:0061458	1.8	2 × 10^−2^									1.7	4 × 10^−2^
Morphogenesis of a branching structure	GO:0001763	1.8	2 × 10^−2^							−2.0	1 × 10^−2^		
Pattern specification process	GO:0007389	1.7	3 × 10^−2^									1.9	2 × 10^−2^
Response to organophosphorus	GO:0046683	1.6	4 × 10^−2^									1.9	3 × 10^−2^
**KEGG Pathways ^2^**													
Nervous system													
Nicotine addiction	ssc05033			−2.1	2 × 10^−8^	−1.8	9 × 10^−3^			1.9	3 × 10^−3^		
Morphine addiction	ssc05032			−1.8	5 × 10^−3^					1.7	3 × 10^−2^		
GABAergic synapse	ssc04727			−1.8	5 × 10^−3^					1.7	3 × 10^−2^		
Oxidative phosphorylation	ssc00190			−1.8	1 × 10^−2^					1.9	2 × 10^−3^	−1.9	2 × 10^−2^
Protein digestion and absorption	ssc04974	1.9	2 × 10^−3^	−1.7	2 × 10^−2^							2.0	2 × 10^−3^
Ribosome	ssc03010	−2.2	2 × 10^−8^	1.9	3 × 10^−2^					2.0	2 × 10^−8^	−2.3	2 × 10^−8^
Long-term potentiation	ssc04720	−1.7	4 × 10^−2^	−1.7	4 × 10^−2^							−1.8	3 × 10^−2^
Immune system													
Graft-versus-host disease	ssc05332	−2.0	9 × 10^−4^									2.3	2 × 10^−8^
Measles	ssc05162	−1.8	2 × 10^−2^									1.7	2 × 10^−2^
Antigen processing and presentation	ssc04612	−1.8	2 × 10^−2^									2.4	2 × 10^−8^
Th17 cell differentiation	ssc04659	−1.7	3 × 10^−2^									1.9	3 × 10^−3^

^1^ Gene Ontology. ^2^ Kyoto Encyclopedia of Genes and Genomes pathway. ^3^ NES > 0 (NES < 0) denotes gene over-expression (under-expression) in control relative to MIA-exposed pigs, pigs exposed to weaning stress relative to not stressed (nursed), and males relative to females.

**Table 2:** Log_2_(fold change) and False Discovery Rate-adjusted *p*-value (LogFC) and FDR, respectively, of the genes in the pituitary gland that presented a significant (FDR-adjusted *p*-value < 0.05) interaction or main effect of maternal immune activation (MIA), weaning, and sex and represent the pathways enriched at FDR < 0.05 and a normalized enrichment score |NES| > 1.6.

Gene	MIA		Weaning		Sex		MIA × Sex		Weaning × Sex		Weaning × MIA	
Symbol	LogFC ^1^	FDR	LogFC	FDR	LogFC	FDR	LogFC	FDR	LogFC	FDR	LogFC	FDR
Neuronal ensheathment												
*Cldn11*	0.7	4 × 10^−1^	0.1	1 × 10^0^	−3.7	3 × 10^−5^	4.7	3 × 10^−5^	−0.3	1 × 10^0^	−3.0	3 × 10^−5^
*Olig2*	0.3	1 × 10^0^	−0.2	1 × 10^0^	−4.7	3 × 10^−5^	4.7	3 × 10^−5^	−0.3	1 × 10^0^	−4.6	1 × 10^−5^
*Mal*	1.2	1 × 10^−2^	0.2	1 × 10^0^	−7.0	4 × 10^−5^	8.8	2 × 10^−5^	−0.8	1 × 10^0^	−6.8	1 × 10^−5^
*Plp1*	1.1	5 × 10^−2^	0.0	1 × 10^0^	−7.8	5 × 10^−5^	8.7	4 × 10^−5^	0.1	1 × 10^0^	−6.3	2 × 10^−5^
Immune system												
*Cxcl10*	−0.2	1 × 10^0^	−0.8	3 × 10^−1^	−1.3	2 × 10^−3^	4.4	6 × 10^−8^	1.4	2 × 10^−1^	2.2	3 × 10^−4^
*Cxcl9*	−2.2	3 × 10^−7^	−0.5	1 × 10^0^	−0.1	1 × 10^0^	1.7	3 × 10^−2^	0.0	1 × 10^0^	2.4	4 × 10^−4^
*Tnfrsf8*	1.9	2 × 10^−5^	−1.9	1 × 10^−5^	−2.1	6 × 10^−8^	2.4	5 × 10^−5^	2.3	5 × 10^−4^	1.9	9 × 10^−3^
*Gdf1*	1.2	1 × 10^−2^	−0.3	1 × 10^0^	−3.3	5 × 10^−8^	4.3	6 × 10^−8^	0.7	1 × 10^0^	−2.1	5 × 10^−4^

^1^ LogFC > 0 (LogFC < 0) denotes (for main and interaction effects) gene over-expression (under-expression) in control relative to MIA-exposed pigs, pigs exposed to weaning stress relative to not stressed (nursed), and males relative to females.

## Data Availability

Data available from the corresponding author on request.

## Supplementary Materials

The following supporting information can be downloaded at: https://www.mdpi.com/article/10.3390/genes14051007/s1. Figure S1. Experimental design scheme. Table S1. Log_2_(fold change) and False Discovery Rate-adjusted *p*-value (LogFC and FDR, respectively) of the genes in the pituitary gland with significant (FDR *p*-value < 0.05 and |LogFC| > 2.5) interaction or main effect of maternal immune activation (MIA), weaning (W), and sex (S); Table S2. Gene Ontology biological processes and Kyoto Encyclopedia of Genes and Genomes pathways enriched (normalized enrichment score NES or ratio > 1.6, FDR *p*-value < 0.05) identified by Gene Set Enrichment Analysis (GSEA) and by Over-representation Analysis (ORA using genes with FDR *p*-value < 0.05) for the interactions and main effects of maternal immune activation (M), weaning (W), and sex (S) in pituitary gland; Table S3. Log_2_(fold change) of the genes in the ensheathment of neurons Kyoto Encyclopedia of Genes and Genomes pathway for the 12 pairwise contrasts between groups characterized by the maternal immune activation, weaning and sex level.

## References

[1] Rymut H.E., Bolt C.R., Caputo M.P., Houser A.K., Antonson A.M., Zimmerman J.D., Villamil M.B., Southey B.R., Rund L.A., Johnson R.W. (2020). Long-Lasting Impact of Maternal Immune Activation and Interaction with a Second Immune Challenge on Pig Behavior. Front. Vet. Sci..

[2] French S.S., Chester E.M., Demas G.E. (2013). Maternal immune activation affects litter success, size and neuroendocrine responses related to behavior in adult offspring. Physiol. Behav..

[3] Knuesel I., Chicha L., Britschgi M., Schobel S.A., Bodmer M., Hellings J.A., Toovey S., Prinssen E.P. (2014). Maternal immune activation and abnormal brain development across CNS disorders. Nat. Rev. Neurol..

[4] Einarsson S., Brandt Y., Lundeheim N., Madej A. (2008). Stress and its influence on reproduction in pigs: A review. Acta Vet. Scand..

[5] Crawley J.N., Heyer W.D., LaSalle J.M. (2016). Autism and Cancer Share Risk Genes, Pathways, and Drug Targets. Trends Genet..

[6] Campbell J.M., Crenshaw J.D., Polo J. (2013). The biological stress of early weaned piglets. J. Anim. Sci. Biotechnol..

[7] Southey B.R., Bolt C.R., Rymut H.E., Keever M.R., Ulanov A.V., Li Z., Rund L.A., Johnson R.W., Rodriguez-Zas S.L. (2021). Impact of Weaning and Maternal Immune Activation on the Metabolism of Pigs. Front. Mol. Biosci..

[8] Maskal J.M., Brito L.F., Duttlinger A.W., Kpodo K.R., McConn B.R., Byrd C.J., Richert B.T., Marchant J.N., Lay D.C., Perry S.D. (2021). Characterizing the postnatal hypothalamic-pituitary-adrenal axis response of in utero heat stressed pigs at 10 and 15 weeks of age. Sci. Rep..

[9] Ji X., Shen Q., Wu P., Chen H., Wang S., Chen D., Yu Y., Guo Z., Wang J., Tang G. (2022). Pituitary-Gland-Based Genes Participates in Intrauterine Growth Restriction in Piglets. Genes.

[10] Ronovsky M., Berger S., Molz B., Berger A., D Pollak D. (2016). Animal models of maternal immune activation in depression research. Curr. Neuropharmacol..

[11] Makris G., Agorastos A., Chrousos G.P., Pervanidou P. (2021). Stress System Activation in Children and Adolescents with Autism Spectrum Disorder. Front. Neurosci..

[12] Southey B.R., Zhang P., Keever M.R., Rymut H.E., Johnson R.W., Sweedler J.V., Rodriguez-Zas S.L. (2021). Effects of maternal immune activation in porcine transcript isoforms of neuropeptide and receptor genes. J. Integr. Neurosci..

[13] Zager A., Andersen M.L., Tufik S., Palermo-Neto J. (2014). Maternal immune activation increases the corticosterone response to acute stress without affecting the hypothalamic monoamine content and sleep patterns in male mice offspring. Neuroimmunomodulation.

[14] Fujitani M., Miyajima H., Otani Y., Liu X. (2022). Maternal and Adult Interleukin-17A Exposure and Autism Spectrum Disorder. Front. Psychiatry.

[15] Rodriguez-Zas S.L., Southey B.R., Rymut H.E., Rund L.A., Johnson R.W. (2022). Hippocampal Changes Elicited by Metabolic and Inflammatory Stressors following Prenatal Maternal Infection. Genes.

[16] Keever-Keigher M.R., Zhang P., Bolt C.R., Rymut H.E., Antonson A.M., Corbett M.P., Houser A.K., Hernandez A.G., Southey B.R., Rund L.A. (2021). Interacting impact of maternal inflammatory response and stress on the amygdala transcriptome of pigs. G3.

[17] Rymut H.E., Rund L.A., Bolt C.R., Villamil M.B., Bender D.E., Southey B.R., Johnson R.W., Rodriguez-Zas S.L. (2021). Biochemistry and Immune Biomarkers Indicate Interacting Effects of Pre- and Postnatal Stressors in Pigs across Sexes. Animals.

[18] Andrews S. A Quality Control Tool for High Throughput Sequence Data. https://www.bioinformatics.babraham.ac.uk/projects/fastqc/.

[19] Pruitt K.D., Tatusova T., Maglott D.R. (2007). NCBI reference sequences (RefSeq): A curated non-redundant sequence database of genomes, transcripts and proteins. Nucleic Acids Res..

[20] Bray N.L., Pimentel H., Melsted P., Pachter L. (2016). Near-optimal probabilistic RNA-seq quantification. Nat. Biotechnol..

[21] Zhang P., Moye L.S., Southey B.R., Dripps I., Sweedler J.V., Pradhan A., Rodriguez-Zas S.L. (2019). Opioid-Induced Hyperalgesia Is Associated with Dysregulation of Circadian Rhythm and Adaptive Immune Pathways in the Mouse Trigeminal Ganglia and Nucleus Accumbens. Mol. Neurobiol..

[22] Robinson M.D., McCarthy D.J., Smyth G.K. (2010). edgeR: A Bioconductor package for differential expression analysis of digital gene expression data. Bioinformatics.

[23] Barrett T., Wilhite S.E., Ledoux P., Evangelista C., Kim I.F., Tomashevsky M., Marshall K.A., Phillippy K.H., Sherman P.M., Holko M. (2013). NCBI GEO: Archive for functional genomics data sets—Update. Nucleic Acids Res..

[24] Kanehisa M., Goto S. (2000). KEGG: Kyoto encyclopedia of genes and genomes. Nucleic Acids Res..

[25] Kanehisa M. (2019). Toward understanding the origin and evolution of cellular organisms. Protein Sci..

[26] Gene Ontology Consortium (2019). The Gene Ontology Resource: 20 years and still GOing strong. Nucleic Acids Res..

[27] Liao Y., Wang J., Jaehnig E.J., Shi Z., Zhang B. (2019). WebGestalt 2019: Gene set analysis toolkit with revamped UIs and APIs. Nucleic Acids Res..

[28] Shannon P., Markiel A., Ozier O., Baliga N.S., Wang J.T., Ramage D., Amin N., Schwikowski B., Ideker T. (2003). Cytoscape: A software environment for integrated models of biomolecular interaction networks. Genome Res..

[29] Szklarczyk D., Gable A.L., Nastou K.C., Lyon D., Kirsch R., Pyysalo S., Doncheva N.T., Legeay M., Fang T., Bork P. (2021). The STRING database in 2021: Customizable protein-protein networks, and functional characterization of user-uploaded gene/measurement sets. Nucleic Acids Res..

[30] Keever M.R., Zhang P., Bolt C.R., Antonson A.M., Rymut H.E., Caputo M.P., Houser A.K., Hernandez A.G., Southey B.R., Rund L.A. (2020). Lasting and sex-dependent impact of maternal immune activation on molecular pathways of the amygdala. Front. Neurosci..

[31] Rymut H.E., Rund L.A., Bolt C.R., Villamil M.B., Southey B.R., Johnson R.W., Rodriguez-Zas S.L. (2021). The Combined Effect of Weaning Stress and Immune Activation during Pig Gestation on Serum Cytokine and Analyte Concentrations. Animals.

[32] Makinodan M., Tatsumi K., Manabe T., Yamauchi T., Makinodan E., Matsuyoshi H., Shimoda S., Noriyama Y., Kishimoto T., Wanaka A. (2008). Maternal immune activation in mice delays myelination and axonal development in the hippocampus of the offspring. J. Neurosci. Res..

[33] Mabuchi Y., Shirasawa N., Sakuma E., Wada I., Horiuchi O., Kikuchi M., Sakamoto A., Herbert D.C., Soji T. (2008). Electron microscopic observations of the anterior pituitary gland: Part I. The neurons in the “transitional zone” of the anterior pituitary gland. Tissue Cell.

[34] Timmons M., Tsokos M., Asab M.A., Seminara S.B., Zirzow G.C., Kaneski C.R., Heiss J.D., van der Knaap M.S., Vanier M.T., Schiffmann R. (2006). Peripheral and central hypomyelination with hypogonadotropic hypogonadism and hypodontia. Neurology.

[35] Page N.F., Gandal M.J., Estes M.L., Cameron S., Buth J., Parhami S., Ramaswami G., Murray K., Amaral D.G., Van de Water J.A. (2021). Alterations in Retrotransposition, Synaptic Connectivity, and Myelination Implicated by Transcriptomic Changes Following Maternal Immune Activation in Nonhuman Primates. Biol. Psychiatry.

[36] Kikusui T., Kiyokawa Y., Mori Y. (2007). Deprivation of mother–pup interaction by early weaning alters myelin formation in male, but not female, ICR mice. Brain Res..

[37] Lee G.A., Lin Y.-K., Lai J.-H., Lo Y.-C., Yang Y.-C.S., Ye S.-Y., Lee C.-J., Wang C.-C., Chiang Y.-H., Tseng S.-H. (2021). Maternal immune activation causes social behavior deficits and hypomyelination in male rat offspring with an autism-like microbiota profile. Brain Sci..

[38] Richetto J., Chesters R., Cattaneo A., Labouesse M.A., Gutierrez A.M.C., Wood T.C., Luoni A., Meyer U., Vernon A., Riva M.A. (2017). Genome-Wide Transcriptional Profiling and Structural Magnetic Resonance Imaging in the Maternal Immune Activation Model of Neurodevelopmental Disorders. Cereb. Cortex.

[39] Zucchi F.C.R., Yao Y., Ilnytskyy Y., Robbins J.C., Soltanpour N., Kovalchuk I., Kovalchuk O., Metz G.A.S. (2014). Lifetime Stress Cumulatively Programs Brain Transcriptome and Impedes Stroke Recovery: Benefit of Sensory Stimulation. PLoS ONE.

[40] Escorcio-Bezerra M.L., Pinto W.B.V.R., Bichuetti D.B., Souza P.V.S., Nunes R.M., Silva L.H.L., Lima K.D.F., Manzano G.M., Oliveira A.S.B., Baeta A.M. (2020). Immune-mediated inflammatory polyneuropathy overlapping Charcot-Marie-Tooth 1B. J. Clin. Neurosci..

[41] Leyrolle Q., Decoeur F., Briere G., Amadieu C., Quadros A.R.A.A., Voytyuk I., Lacabanne C., Benmamar-Badel A., Bourel J., Aubert A. (2021). Maternal dietary omega-3 deficiency worsens the deleterious effects of prenatal inflammation on the gut-brain axis in the offspring across lifetime. Neuropsychopharmacology.

[42] Sunman B., Yurdakök K., Kocer-Gumusel B., Özyüncü Ö., Akbıyık F., Balcı A., Özkemahlı G., Erkekoğlu P., Yurdakök M. (2019). Prenatal bisphenol a and phthalate exposure are risk factors for male reproductive system development and cord blood sex hormone levels. Reprod. Toxicol..

[43] Nandwana V., Nandwana N.K., Das Y., Saito M., Panda T., Das S., Almaguel F., Hosmane N.S., Das B.C. (2022). The Role of Microbiome in Brain Development and Neurodegenerative Diseases. Molecules.

[44] Reshetnikov V.V., Kisaretova P.E., Bondar N.P. (2022). Transcriptome Alterations Caused by Social Defeat Stress of Various Durations in Mice and Its Relevance to Depression and Posttraumatic Stress Disorder in Humans: A Meta-Analysis. Int. J. Mol. Sci..

[45] Roshan-Milani S., Seyyedabadi B., Saboory E., Parsamanesh N., Mehranfard N. (2021). Prenatal stress and increased susceptibility to anxiety-like behaviors: Role of neuroinflammation and balance between GABAergic and glutamatergic transmission. Stress.

[46] Rymut H.E., Rund L.A., Southey B.R., Johnson R.W., Rodriguez-Zas S.L. (2022). Terpenoid Backbone Biosynthesis among Pig Hippocampal Pathways Impacted by Stressors. Genes.

[47] Gorlova A.V., Pavlov D.A., Ushakova V.M., Zubkov E.A., Zorkina Y.A., Morozova A.Y., Inozemtsev A.N., Chekhonin V.P. (2020). The Induction of a Depression-Like State by Chronic Exposure to Ultrasound in Rats Is Accompanied by a Reduction in Gene Expression of GABAA-Receptor Subunits in the Brain. Neurochem. J..

[48] Mozhui K., Karlsson R.-M., Kash T.L., Ihne J., Norcross M., Patel S., Farrell M.R., Hill E.E., Graybeal C., Martin K.P. (2010). Strain Differences in Stress Responsivity Are Associated with Divergent Amygdala Gene Expression and Glutamate-Mediated Neuronal Excitability. J. Neurosci..

[49] Rymut H.E., Rund L.A., Southey B.R., Johnson R.W., Sweedler J.V., Rodriguez-Zas S.L. (2022). Prefrontal Cortex Response to Prenatal Insult and Postnatal Opioid Exposure. Genes.

[50] Dorshkind K., Horseman N.D. (2001). Anterior pituitary hormones, stress, and immune system homeostasis. BioEssays.

[51] Anderson A., Genaro-Mattos T.C., Allen L.B., Koczok K., Korade Z., Mirnics K. (2021). Interaction of maternal immune activation and genetic interneuronal inhibition. Brain Res..

[52] Luo W., Fang M., Xu H., Xing H., Nie Q. (2015). Transcriptome comparison in the pituitary–adrenal axis between Beagle and Chinese Field dogs after chronic stress exposure. Anim. Genet..

[53] Konishi H., Ogawa T., Nakagomi S., Inoue K., Tohyama M., Kiyama H. (2010). Id1, Id2 and Id3 are induced in rat melanotrophs of the pituitary gland by dopamine suppression under continuous stress. Neuroscience.

[54] Narayan S., Head S.R., Gilmartin T.J., Dean B., Thomas E.A. (2009). Evidence for disruption of sphingolipid metabolism in schizophrenia. J. Neurosci. Res..

[55] Gebicke-Haerter P.J., Leonardi-Essmann F., Haerter J.O., Rossner M.J., Falkai P., Schmitt A., Raabe F.J. (2021). Differential gene regulation in the anterior cingulate cortex and superior temporal cortex in schizophrenia: A molecular network approach. Schizophr. Res..

