# Supplementary material for: Genes Participating in the Ensheathment of Neurons Are Affected by Postnatal Stress and Maternal Immune Activation in the Pituitary Gland
Source: Genes (Basel). 2023 Apr 28;14(5):1007. doi: 10.3390/genes14051007 (PMC10218591; doi:10.3390/genes14051007)
Supplement: Supplementary file 1 [file genes-14-01007-s001.zip › PituD22_SupplementalTables_Apr232023.pdf]

Supplemental Materials

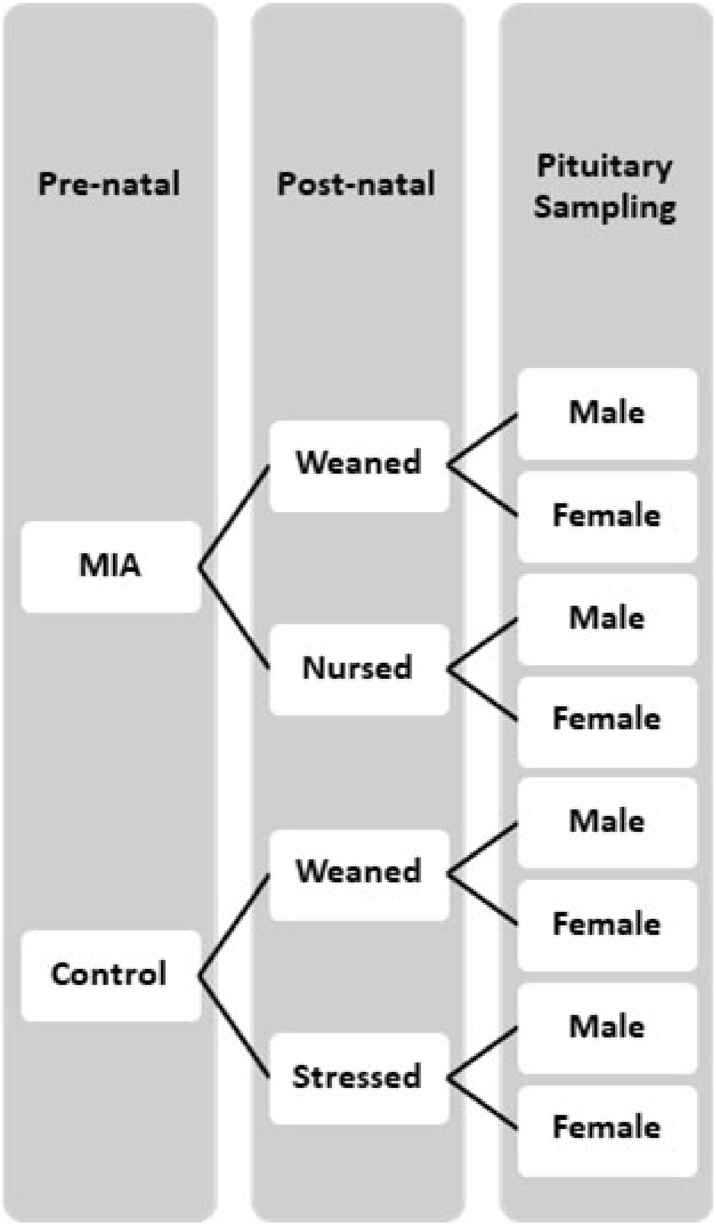

Supplemental Figure S1. Experimental design scheme.

Supplemental Table S1. Log_2(fold change) and False Discovery Rate-adjusted *p*-value (LogFC) and FDR, respectively) of the genes in the pituitary gland with significant (FDR *p*-value < 0.05 and ∣LogFC∣ > 2.5) interaction or main effect of maternal immune activation (MIA), weaning, and sex.

Effect	Gene Symbol	LogFC[1]	FDR
Weaning-by-Sex	*Sst*	6.22	5×10^{-8}
Weaning-by-Sex	*Npy*	5.35	4×10^{-8}
Weaning-by-Sex	*Tac1*	4.76	3×10^{-8}
Weaning-by-Sex	*Col11a2*	4.59	1×10^{-8}
Weaning-by-Sex	*Agrp*	4.66	7×10^{-8}
Weaning-by-Sex	*Gabrq*	4.51	8×10^{-8}
Weaning-by-Sex	*Camkv*	4.4	1×10^{-8}
Weaning-by-Sex	*Loc110256816*	4.22	1×10^{-8}
Weaning-by-Sex	*Fndc9*	4.04	7×10^{-8}
Weaning-by-Sex	*Loc100511183*	3.86	4×10^{-8}
Weaning-by-Sex	*Nptx1*	3.87	4×10^{-8}
Weaning-by-Sex	*Gtsf1*	-3.97	2×10^{-8}
Weaning-by-Sex	*Slc6a7*	3.65	1×10^{-8}
Weaning-by-Sex	*Loc100158003*	3.64	8×10^{-8}
Weaning-by-Sex	*Gal*	3.45	1×10^{-8}
Weaning-by-Sex	*Comp*	3.69	1×10^{-8}
Weaning-by-Sex	*Cbln4*	3.64	1×10^{-8}
Weaning-by-Sex	*Loc110256043*	3.32	1×10^{-8}
Weaning-by-Sex	*Loc102161214*	4.07	4×10^{-8}
Weaning-by-Sex	*Atp5pd*	3.22	7×10^{-8}
Weaning-by-Sex	*Slc5a5*	3.23	2×10^{-8}
Weaning-by-Sex	*Odf3b*	3.36	4×10^{-8}
Weaning-by-Sex	*Grin1*	3.08	8×10^{-8}
Weaning-by-Sex	*Cacna2d3*	3.12	9×10^{-8}
Weaning-by-Sex	*Sap25*	3.06	9×10^{-8}
Weaning-by-Sex	*Draxin*	3.24	9×10^{-7}
Weaning-by-Sex	*Glra2*	3.05	2×10^{-7}
Weaning-by-Sex	*Calb2*	3.04	2×10^{-7}
Weaning-by-Sex	*Cpne6*	3.15	3×10^{-7}
Weaning-by-Sex	*Loc100525452*	3.13	3×10^{-7}
Weaning-by-Sex	*Ttr*	3.2	3×10^{-7}
Weaning-by-Sex	*Msmp*	-3.20	3×10^{-7}
Weaning-by-Sex	*Vsnl1*	2.9	5×10^{-7}
Weaning-by-Sex	*Loc100152209*	-2.87	6×10^{-7}
Weaning-by-Sex	*Slc22a2*	2.97	6×10^{-7}
Weaning-by-Sex	*Sult1a3*	2.89	6×10^{-7}

Weaning-by-Sex	*Shisa9*	2.96	7×10^{-7}
Weaning-by-Sex	*Osr1*	2.9	1×10^{-6}
Weaning-by-Sex	*Notum*	2.85	2×10^{-6}
Weaning-by-Sex	*Slc13a4*	2.69	3×10^{-6}
Weaning-by-Sex	*Loc110258721*	2.98	4×10^{-6}
Weaning-by-Sex	*Hs6st3*	2.74	5×10^{-6}
Weaning-by-Sex	*Loc110258908*	2.81	7×10^{-6}
Weaning-by-Sex	*Cartpt*	2.61	7×10^{-6}
Weaning-by-Sex	*Rn5-8s*	2.56	1×10^{-5}
Weaning-by-Sex	*Npw*	2.68	2×10^{-5}
Weaning-by-Sex	*Hdc*	2.67	2×10^{-5}
Weaning-by-Sex	*C3h16orf89*	2.56	2×10^{-5}
Weaning-by-Sex	*Nxph1*	2.7	2×10^{-5}
Weaning-by-Sex	*Tmem196*	2.76	3×10^{-5}
Weaning-by-Sex	*Gjb2*	2.69	3×10^{-5}
Weaning-by-Sex	*Zic5*	2.81	3×10^{-5}
Weaning-by-Sex	*Eif1ay*	-4.91	4×10^{-5}
Weaning-by-Sex	*Ngb*	2.51	5×10^{-5}
Weaning-by-Sex	*Cbln1*	2.62	2×10^{-4}
Weaning-by-Sex	*Loc110259430*	-2.67	1×10^{-3}
Weaning-by-Sex	*Loc110257903*	3.05	3×10^{-3}
Weaning-by-Sex	*Loc110257905*	-3.66	3×10^{-3}
Weaning-by-Sex	*Loc100624590*	-3.45	8×10^{-3}
MIA-by-Weaning	*Mobp*	-7.66	6×10^{-8}
MIA-by-Weaning	*Mbp*	-7.51	8×10^{-8}
MIA-by-Weaning	*Ermn*	-7.11	2×10^{-8}
MIA-by-Weaning	*Hapln2*	-7.55	2×10^{-8}
MIA-by-Weaning	*Avp*	-6.75	3×10^{-8}
MIA-by-Weaning	*Mag*	-6.55	3×10^{-8}
MIA-by-Weaning	*Pou3f3*	-6.83	3×10^{-8}
MIA-by-Weaning	*Mal*	-6.81	1×10^{-8}
MIA-by-Weaning	*Plp1*	-6.32	2×10^{-8}
MIA-by-Weaning	*Mog*	-6.36	3×10^{-8}
MIA-by-Weaning	*Gjb1*	-6.79	2×10^{-8}
MIA-by-Weaning	*Slc14a1*	-6.25	4×10^{-8}
MIA-by-Weaning	*Nkx6-2*	-6.23	4×10^{-8}
MIA-by-Weaning	*Gjc2*	-6.08	5×10^{-8}
MIA-by-Weaning	*Mlc1*	-5.94	1×10^{-8}
MIA-by-Weaning	*Grm3*	-6.09	2×10^{-8}
MIA-by-Weaning	*Fa2h*	-5.81	3×10^{-8}

MIA-by-Weaning	*Gabrq*	6.65	3×10^{-8}
MIA-by-Weaning	*Ugt8*	-5.52	3×10^{-8}
MIA-by-Weaning	*Adamts4*	-5.54	7×10^{-8}
MIA-by-Weaning	*Nphs1*	-5.65	3×10^{-8}
MIA-by-Weaning	*Ttyh1*	-5.41	6×10^{-8}
MIA-by-Weaning	*Tmem88b*	-5.45	9×10^{-8}
MIA-by-Weaning	*Cmtm5*	-5.49	2×10^{-8}
MIA-by-Weaning	*Fabp7*	-5.28	6×10^{-8}
MIA-by-Weaning	*Tf*	-5.19	1×10^{-8}
MIA-by-Weaning	*Sox10*	-5.13	9×10^{-8}
MIA-by-Weaning	*Loc110256043*	5.2	3×10^{-8}
MIA-by-Weaning	*Sgk2*	-5.16	4×10^{-8}
MIA-by-Weaning	*Tubb4a*	-4.86	7×10^{-8}
MIA-by-Weaning	*Fut2*	-5.02	9×10^{-8}
MIA-by-Weaning	*Elfn2*	-4.89	1×10^{-8}
MIA-by-Weaning	*Olig1*	-4.72	1×10^{-8}
MIA-by-Weaning	*Loc102161214*	5.76	2×10^{-8}
MIA-by-Weaning	*Slc17a7*	-4.73	3×10^{-8}
MIA-by-Weaning	*Igsf21*	-5.07	4×10^{-8}
MIA-by-Weaning	*Opalin*	-4.64	5×10^{-8}
MIA-by-Weaning	*Slc5a5*	4.95	9×10^{-8}
MIA-by-Weaning	*Bcan*	-4.6	1×10^{-8}
MIA-by-Weaning	*Slc6a1*	-4.61	1×10^{-8}
MIA-by-Weaning	*Olig2*	-4.63	1×10^{-8}
MIA-by-Weaning	*Plxnb3*	-4.61	1×10^{-8}
MIA-by-Weaning	*Slc1a2*	-4.55	2×10^{-8}
MIA-by-Weaning	*Synpo2l*	-4.59	5×10^{-8}
MIA-by-Weaning	*Sh3tc2*	-4.7	5×10^{-8}
MIA-by-Weaning	*Stum*	-4.51	7×10^{-8}
MIA-by-Weaning	*Gfap*	-4.44	1×10^{-8}
MIA-by-Weaning	*Agrp*	4.89	2×10^{-8}
MIA-by-Weaning	*Dact2*	-4.62	2×10^{-8}
MIA-by-Weaning	*Fam131b*	-4.35	1×10^{-8}
MIA-by-Weaning	*C14h10orf90*	-4.51	1×10^{-8}
MIA-by-Weaning	*Kcnj10*	-4.31	1×10^{-8}
MIA-by-Weaning	*Loc110261683*	-4.52	2×10^{-8}
MIA-by-Weaning	*Atp10b*	-4.35	4×10^{-8}
MIA-by-Weaning	*Gpr37l1*	-4.21	9×10^{-8}
MIA-by-Weaning	*C4h8orf46*	-4.03	2×10^{-8}
MIA-by-Weaning	*Oxt*	-4.00	2×10^{-8}

MIA-by-Weaning	*Ppp1r14a*	-3.99	2×10^{-8}
MIA-by-Weaning	*Adora1*	-3.93	1×10^{-8}
MIA-by-Weaning	*Rab33a*	-3.93	3×10^{-8}
MIA-by-Weaning	*Loc100620470*	-3.83	8×10^{-8}
MIA-by-Weaning	*Gpr17*	-3.74	2×10^{-8}
MIA-by-Weaning	*Fam135b*	-3.75	3×10^{-8}
MIA-by-Weaning	*Acsbg1*	-3.64	6×10^{-8}
MIA-by-Weaning	*Mpped1*	-3.67	7×10^{-8}
MIA-by-Weaning	*Ddn*	-3.63	9×10^{-8}
MIA-by-Weaning	*Loc100624063*	-3.84	1×10^{-8}
MIA-by-Weaning	*S1pr5*	-3.63	1×10^{-8}
MIA-by-Weaning	*Foxg1*	-3.68	4×10^{-8}
MIA-by-Weaning	*Slc22a2*	3.79	5×10^{-8}
MIA-by-Weaning	*Adap1*	-3.45	1×10^{-8}
MIA-by-Weaning	*Gad1*	-3.48	1×10^{-8}
MIA-by-Weaning	*Pllp*	-3.4	2×10^{-8}
MIA-by-Weaning	*Neurod2*	-3.51	2×10^{-8}
MIA-by-Weaning	*Myrf*	-3.38	3×10^{-8}
MIA-by-Weaning	*Loc110257903*	4.04	5×10^{-8}
MIA-by-Weaning	*Cnp*	-3.29	8×10^{-8}
MIA-by-Weaning	*Gal3st1*	-3.31	8×10^{-8}
MIA-by-Weaning	*Ecel1*	3.34	1×10^{-8}
MIA-by-Weaning	*Efhd1*	-3.28	1×10^{-8}
MIA-by-Weaning	*Unc13c*	-3.30	2×10^{-8}
MIA-by-Weaning	*Grin2a*	-3.27	5×10^{-8}
MIA-by-Weaning	*Ca14*	-3.22	6×10^{-8}
MIA-by-Weaning	*Egr3*	-3.26	7×10^{-8}
MIA-by-Weaning	*Pygm*	-3.08	2×10^{-8}
MIA-by-Weaning	*Sult1a3*	3.18	2×10^{-8}
MIA-by-Weaning	*Loc100512593*	-3.17	2×10^{-8}
MIA-by-Weaning	*Slc1a3*	-3.04	2×10^{-8}
MIA-by-Weaning	*Cldn11*	-3.03	3×10^{-8}
MIA-by-Weaning	*Ubd*	3.57	3×10^{-8}
MIA-by-Weaning	*Col2a1*	3.07	3×10^{-8}
MIA-by-Weaning	*Rab7b*	-3.06	3×10^{-8}
MIA-by-Weaning	*Gbp1*	3.05	3×10^{-8}
MIA-by-Weaning	*Cabp1*	-3.05	3×10^{-8}
MIA-by-Weaning	*Camk2a*	-3.00	4×10^{-8}
MIA-by-Weaning	*Apln*	-3.01	4×10^{-8}
MIA-by-Weaning	*Paqr6*	-2.99	4×10^{-8}

MIA-by-Weaning	*Dmbt1*	3.34	4×10^{-8}
MIA-by-Weaning	*Cdh19*	-3.24	4×10^{-8}
MIA-by-Weaning	*Fgfr3*	-2.99	5×10^{-8}
MIA-by-Weaning	*Scd*	-2.96	6×10^{-8}
MIA-by-Weaning	*Gpr50*	3.42	6×10^{-8}
MIA-by-Weaning	*Egr4*	-3.16	7×10^{-8}
MIA-by-Weaning	*Prss56*	3.03	7×10^{-8}
MIA-by-Weaning	*Comp*	3.19	8×10^{-8}
MIA-by-Weaning	*Tmem155*	-3.10	8×10^{-8}
MIA-by-Weaning	*Mfsd2a*	-2.95	8×10^{-8}
MIA-by-Weaning	*Mgat5b*	-3.03	9×10^{-8}
MIA-by-Weaning	*Nrgn*	-2.89	1×10^{-7}
MIA-by-Weaning	*Slc5a11*	-3.01	1×10^{-7}
MIA-by-Weaning	*Kcna2*	-2.91	1×10^{-7}
MIA-by-Weaning	*Stmn4*	-2.88	2×10^{-7}
MIA-by-Weaning	*Th*	-3.04	2×10^{-7}
MIA-by-Weaning	*Loc106505443*	3.64	2×10^{-7}
MIA-by-Weaning	*Wnt7b*	-2.98	2×10^{-7}
MIA-by-Weaning	*Snx22*	-2.88	2×10^{-7}
MIA-by-Weaning	*Pou3f2*	-2.9	2×10^{-7}
MIA-by-Weaning	*Arhgdig*	-2.97	2×10^{-7}
MIA-by-Weaning	*Fabp3*	-2.87	2×10^{-7}
MIA-by-Weaning	*Chrm1*	-2.93	2×10^{-7}
MIA-by-Weaning	*Shmt1*	-2.85	3×10^{-7}
MIA-by-Weaning	*Sox21*	-3.08	3×10^{-7}
MIA-by-Weaning	*Shank1*	-2.82	3×10^{-7}
MIA-by-Weaning	*Aqp4*	-2.81	3×10^{-7}
MIA-by-Weaning	*Evi2a*	-2.81	4×10^{-7}
MIA-by-Weaning	*Tpo*	2.85	6×10^{-7}
MIA-by-Weaning	*Lhx9*	3.09	6×10^{-7}
MIA-by-Weaning	*Osr1*	2.91	7×10^{-7}
MIA-by-Weaning	*Rbfox3*	-2.79	7×10^{-7}
MIA-by-Weaning	*Bcas1*	-2.72	9×10^{-7}
MIA-by-Weaning	*Adcy1*	-2.71	1×10^{-6}
MIA-by-Weaning	*Lingo1*	-2.70	1×10^{-6}
MIA-by-Weaning	*Ttr*	-2.91	2×10^{-6}
MIA-by-Weaning	*St8sia6*	2.7	2×10^{-6}
MIA-by-Weaning	*Prima1*	-2.69	2×10^{-6}
MIA-by-Weaning	*Tac1*	2.72	2×10^{-6}
MIA-by-Weaning	*F5*	-2.66	2×10^{-6}

MIA-by-Weaning	*Rasgrp1*	-2.69	2×10^{-6}
MIA-by-Weaning	*Fezf1*	2.71	3×10^{-6}
MIA-by-Weaning	*Gabre*	2.66	3×10^{-6}
MIA-by-Weaning	*Slc1a6*	-2.67	3×10^{-6}
MIA-by-Weaning	*Grin1*	-2.63	4×10^{-6}
MIA-by-Weaning	*Dio3*	2.71	4×10^{-6}
MIA-by-Weaning	*Ca4*	-2.73	4×10^{-6}
MIA-by-Weaning	*Prr18*	-2.58	5×10^{-6}
MIA-by-Weaning	*Ncan*	-2.57	5×10^{-6}
MIA-by-Weaning	*Ltc4s*	-2.71	5×10^{-6}
MIA-by-Weaning	*Cecr6*	-2.61	5×10^{-6}
MIA-by-Weaning	*Mfrp*	2.92	6×10^{-6}
MIA-by-Weaning	*Arc*	-2.56	6×10^{-6}
MIA-by-Weaning	*Loc106510131*	-2.55	7×10^{-6}
MIA-by-Weaning	*Omg*	-2.54	7×10^{-6}
MIA-by-Weaning	*Cldn19*	-3.50	7×10^{-6}
MIA-by-Weaning	*Kcne4*	-2.73	8×10^{-6}
MIA-by-Weaning	*Loc110257701*	2.76	9×10^{-6}
MIA-by-Weaning	*Kcnip1*	-2.61	1×10^{-5}
MIA-by-Weaning	*Ambn*	2.66	1×10^{-5}
MIA-by-Weaning	*Rprml*	-2.55	1×10^{-5}
MIA-by-Weaning	*Gfra3*	2.55	1×10^{-5}
MIA-by-Weaning	*Loc100157711*	-2.55	1×10^{-5}
MIA-by-Weaning	*Nlrc5*	2.51	1×10^{-5}
MIA-by-Weaning	*Hdc*	2.69	1×10^{-5}
MIA-by-Weaning	*Gal*	2.6	2×10^{-5}
MIA-by-Weaning	*Pdlim4*	-2.54	2×10^{-5}
MIA-by-Weaning	*Slc6a7*	-2.52	2×10^{-5}
MIA-by-Weaning	*Loc110257816*	2.56	2×10^{-5}
MIA-by-Weaning	*Lpar3*	2.91	2×10^{-5}
MIA-by-Weaning	*T*	2.64	6×10^{-5}
MIA-by-Weaning	*Uts2r*	2.55	1×10^{-4}
MIA-by-Weaning	*Loc100038328*	3.20	4×10^{-2}
MIA-by-Sex	*Hapln2*	12.00	3×10^{-8}
MIA-by-Sex	*Gjb1*	11.72	3×10^{-8}
MIA-by-Sex	*Gfap*	9.91	7×10^{-8}
MIA-by-Sex	*Mbp*	9.38	1×10^{-8}
MIA-by-Sex	*Tf*	9.34	5×10^{-8}
MIA-by-Sex	*Ermn*	9.02	1×10^{-8}
MIA-by-Sex	*Mobp*	8.88	1×10^{-8}

MIA-by-Sex	*Plp1*	8.73	4×10^{-8}
MIA-by-Sex	*Loc110261683*	8.96	2×10^{-8}
MIA-by-Sex	*Mog*	8.76	1×10^{-8}
MIA-by-Sex	*Mal*	8.75	2×10^{-8}
MIA-by-Sex	*Gjc2*	8.15	2×10^{-8}
MIA-by-Sex	*Fa2h*	7.99	2×10^{-8}
MIA-by-Sex	*Cmtm5*	7.98	2×10^{-8}
MIA-by-Sex	*Mag*	7.61	5×10^{-8}
MIA-by-Sex	*Agt*	7.40	3×10^{-8}
MIA-by-Sex	*Nkx6-1*	7.69	4×10^{-8}
MIA-by-Sex	*Ugt8*	7.38	5×10^{-8}
MIA-by-Sex	*Nkx6-2*	7.39	1×10^{-8}
MIA-by-Sex	*C14h10orf105*	7.23	5×10^{-8}
MIA-by-Sex	*Tmem88b*	7.12	1×10^{-8}
MIA-by-Sex	*Paqr6*	6.84	4×10^{-8}
MIA-by-Sex	*Tubb4a*	6.82	6×10^{-8}
MIA-by-Sex	*Sh3tc2*	6.96	1×10^{-8}
MIA-by-Sex	*Gpr37l1*	6.71	9×10^{-8}
MIA-by-Sex	*Slc14a1*	6.90	1×10^{-8}
MIA-by-Sex	*Efhd1*	6.64	5×10^{-8}
MIA-by-Sex	*Mlc1*	6.61	8×10^{-8}
MIA-by-Sex	*Sgk2*	6.73	1×10^{-8}
MIA-by-Sex	*Synpo2l*	6.50	4×10^{-8}
MIA-by-Sex	*Evi2a*	6.36	5×10^{-8}
MIA-by-Sex	*Sox10*	6.25	1×10^{-8}
MIA-by-Sex	*Slc5a11*	6.33	2×10^{-8}
MIA-by-Sex	*Ppp1r14a*	6.08	2×10^{-8}
MIA-by-Sex	*Apod*	5.96	2×10^{-8}
MIA-by-Sex	*Gal*	6.07	7×10^{-8}
MIA-by-Sex	*Rab7b*	6.01	7×10^{-8}
MIA-by-Sex	*Irx1*	6.41	1×10^{-8}
MIA-by-Sex	*Opalin*	5.89	1×10^{-8}
MIA-by-Sex	*Kctd4*	6.12	6×10^{-8}
MIA-by-Sex	*C14h10orf90*	5.9	1×10^{-8}
MIA-by-Sex	*Adamts4*	5.8	2×10^{-8}
MIA-by-Sex	*Zic1*	5.74	1×10^{-8}
MIA-by-Sex	*Olig1*	5.63	2×10^{-8}
MIA-by-Sex	*Fabp7*	5.54	1×10^{-8}
MIA-by-Sex	*Gabrq*	6.53	2×10^{-8}
MIA-by-Sex	*Ttyh1*	5.48	5×10^{-8}

MIA-by-Sex	*Slc17a7*	-5.36	9×10^{-8}
MIA-by-Sex	*S1pr5*	5.47	1×10^{-8}
MIA-by-Sex	*Pdlim4*	5.52	1×10^{-8}
MIA-by-Sex	*Pou3f3*	5.33	9×10^{-8}
MIA-by-Sex	*Nkd1*	5.19	1×10^{-8}
MIA-by-Sex	*Snx22*	5.14	2×10^{-8}
MIA-by-Sex	*Cnmd*	5.17	3×10^{-8}
MIA-by-Sex	*Kcnip1*	5.23	4×10^{-8}
MIA-by-Sex	*Atp10b*	5.25	4×10^{-8}
MIA-by-Sex	*Zic5*	5.40	6×10^{-8}
MIA-by-Sex	*Loc110256043*	5.12	6×10^{-8}
MIA-by-Sex	*Fam135b*	5.06	4×10^{-8}
MIA-by-Sex	*Aspa*	5.07	6×10^{-8}
MIA-by-Sex	*Ndp*	5.07	6×10^{-8}
MIA-by-Sex	*Kcnj10*	4.93	7×10^{-8}
MIA-by-Sex	*Calb2*	5.01	1×10^{-8}
MIA-by-Sex	*Fez1*	4.86	2×10^{-8}
MIA-by-Sex	*Flt3*	5.04	2×10^{-8}
MIA-by-Sex	*Emilin2*	4.88	3×10^{-8}
MIA-by-Sex	*Bcan*	4.83	4×10^{-8}
MIA-by-Sex	*Cldn11*	4.71	3×10^{-8}
MIA-by-Sex	*Olig2*	4.74	3×10^{-8}
MIA-by-Sex	*Loc110255851*	4.90	4×10^{-8}
MIA-by-Sex	*Aplnr*	4.69	4×10^{-8}
MIA-by-Sex	*Apoe*	4.57	3×10^{-8}
MIA-by-Sex	*Myrf*	4.57	3×10^{-8}
MIA-by-Sex	*Dthd1*	4.75	7×10^{-8}
MIA-by-Sex	*Rflnb*	4.52	7×10^{-8}
MIA-by-Sex	*Aqp4*	4.49	1×10^{-8}
MIA-by-Sex	*Gal3st1*	4.5	1×10^{-8}
MIA-by-Sex	*Plxnb3*	4.51	1×10^{-8}
MIA-by-Sex	*Homer3*	4.47	2×10^{-8}
MIA-by-Sex	*Hepacam*	4.46	2×10^{-8}
MIA-by-Sex	*Ca14*	4.55	2×10^{-8}
MIA-by-Sex	*Cnp*	4.43	3×10^{-8}
MIA-by-Sex	*Pla2r1*	4.44	4×10^{-8}
MIA-by-Sex	*Pllp*	4.41	4×10^{-8}
MIA-by-Sex	*Comp*	5.06	5×10^{-8}
MIA-by-Sex	*Cxcl10*	4.43	6×10^{-8}
MIA-by-Sex	*Pdyn*	4.44	8×10^{-8}

MIA-by-Sex	*Slc5a5*	4.61	2×10^{-8}
MIA-by-Sex	*Gdf1*	4.25	6×10^{-8}
MIA-by-Sex	*Elfn2*	4.25	9×10^{-8}
MIA-by-Sex	*Col11a2*	4.28	9×10^{-8}
MIA-by-Sex	*Lcat*	4.36	1×10^{-8}
MIA-by-Sex	*Cp*	4.24	1×10^{-8}
MIA-by-Sex	*Slc1a6*	4.28	1×10^{-8}
MIA-by-Sex	*Lgi4*	4.2	2×10^{-8}
MIA-by-Sex	*Grm3*	4.50	2×10^{-8}
MIA-by-Sex	*Smoc1*	4.18	2×10^{-8}
MIA-by-Sex	*Rab33a*	4.22	2×10^{-8}
MIA-by-Sex	*Ido1*	4.45	3×10^{-8}
MIA-by-Sex	*Ada*	4.19	3×10^{-8}
MIA-by-Sex	*Sox21*	4.43	3×10^{-8}
MIA-by-Sex	*Ttyh2*	4.12	4×10^{-8}
MIA-by-Sex	*Cbr3*	4.41	5×10^{-8}
MIA-by-Sex	*Cldn10*	4.19	5×10^{-8}
MIA-by-Sex	*Cryab*	4.1	6×10^{-8}
MIA-by-Sex	*Apln*	4.11	6×10^{-8}
MIA-by-Sex	*Th*	4.54	9×10^{-8}
MIA-by-Sex	*Cpxm2*	4.04	2×10^{-8}
MIA-by-Sex	*Erbb3*	4.03	2×10^{-8}
MIA-by-Sex	*Fgfr4*	4.15	3×10^{-8}
MIA-by-Sex	*S100a1*	3.96	5×10^{-8}
MIA-by-Sex	*Plekhb1*	3.95	5×10^{-8}
MIA-by-Sex	*S100a6*	3.98	5×10^{-8}
MIA-by-Sex	*Sult1a3*	4.03	5×10^{-8}
MIA-by-Sex	*C4h8orf46*	3.96	6×10^{-8}
MIA-by-Sex	*Ddn*	-3.89	8×10^{-8}
MIA-by-Sex	*Mmp28*	3.93	1×10^{-8}
MIA-by-Sex	*Zic2*	3.97	1×10^{-8}
MIA-by-Sex	*Fam159b*	3.99	2×10^{-8}
MIA-by-Sex	*Aif1l*	3.90	2×10^{-8}
MIA-by-Sex	*Gpr17*	3.89	2×10^{-8}
MIA-by-Sex	*Cers1*	3.84	3×10^{-8}
MIA-by-Sex	*Prr18*	3.82	4×10^{-8}
MIA-by-Sex	*Gfra3*	3.86	4×10^{-8}
MIA-by-Sex	*Prima1*	3.83	5×10^{-8}
MIA-by-Sex	*Nphs1*	-3.83	6×10^{-8}
MIA-by-Sex	*Map6d1*	3.77	9×10^{-8}

MIA-by-Sex	*Slc2a5*	3.81	9×10^{-8}
MIA-by-Sex	*Kcna6*	3.74	1×10^{-8}
MIA-by-Sex	*Gpr37*	3.74	1×10^{-8}
MIA-by-Sex	*Adap1*	3.75	1×10^{-8}
MIA-by-Sex	*Foxg1*	-3.85	2×10^{-8}
MIA-by-Sex	*Omg*	3.71	2×10^{-8}
MIA-by-Sex	*Loc110257903*	4.41	4×10^{-8}
MIA-by-Sex	*Lrrc71*	3.74	4×10^{-8}
MIA-by-Sex	*Fabp3*	3.66	5×10^{-8}
MIA-by-Sex	*Ctnna3*	3.68	7×10^{-8}
MIA-by-Sex	*Stmn4*	3.62	7×10^{-8}
MIA-by-Sex	*Sbspon*	3.71	8×10^{-8}
MIA-by-Sex	*Smox*	3.57	2×10^{-8}
MIA-by-Sex	*Neurod2*	-3.62	3×10^{-8}
MIA-by-Sex	*Rsad2*	3.52	4×10^{-8}
MIA-by-Sex	*Tmem63a*	3.46	8×10^{-8}
MIA-by-Sex	*Gpd1*	3.45	9×10^{-8}
MIA-by-Sex	*Gbp1*	3.43	1×10^{-8}
MIA-by-Sex	*Tmem144*	3.41	2×10^{-8}
MIA-by-Sex	*Ubd*	3.77	2×10^{-8}
MIA-by-Sex	*Chn2*	3.40	2×10^{-8}
MIA-by-Sex	*Dbndd2*	3.37	3×10^{-8}
MIA-by-Sex	*Loc100156167*	3.6	3×10^{-8}
MIA-by-Sex	*Loc100524517*	3.47	4×10^{-8}
MIA-by-Sex	*Daam2*	3.33	4×10^{-8}
MIA-by-Sex	*Slc18a2*	3.45	4×10^{-8}
MIA-by-Sex	*Neu4*	3.36	5×10^{-8}
MIA-by-Sex	*Grin2a*	-3.4	5×10^{-8}
MIA-by-Sex	*Sdcbp2*	3.41	5×10^{-8}
MIA-by-Sex	*Plac8*	3.36	5×10^{-8}
MIA-by-Sex	*Fam131b*	3.33	5×10^{-8}
MIA-by-Sex	*Actc1*	3.43	5×10^{-8}
MIA-by-Sex	*Lsp1*	3.31	6×10^{-8}
MIA-by-Sex	*Smim5*	3.34	7×10^{-8}
MIA-by-Sex	*Cd38*	3.32	7×10^{-8}
MIA-by-Sex	*Slc16a4*	3.42	9×10^{-8}
MIA-by-Sex	*Bfsp2*	3.39	9×10^{-8}
MIA-by-Sex	*Ecel1*	3.31	9×10^{-8}
MIA-by-Sex	*Col2a1*	3.28	1×10^{-8}
MIA-by-Sex	*C2*	3.29	1×10^{-8}

MIA-by-Sex	*Ndrg1*	3.23	2×10^{-8}
MIA-by-Sex	*Shmt1*	3.24	2×10^{-8}
MIA-by-Sex	*Entpd2*	3.30	2×10^{-8}
MIA-by-Sex	*Rasgrp1*	-3.24	2×10^{-8}
MIA-by-Sex	*Dhrs9*	3.49	2×10^{-8}
MIA-by-Sex	*Osr1*	3.36	2×10^{-8}
MIA-by-Sex	*Coro2b*	3.20	2×10^{-8}
MIA-by-Sex	*B4galnt1*	3.22	2×10^{-8}
MIA-by-Sex	*Isg15*	3.19	3×10^{-8}
MIA-by-Sex	*Kcnk10*	3.19	3×10^{-8}
MIA-by-Sex	*Slc22a2*	3.42	4×10^{-8}
MIA-by-Sex	*Tesk2*	3.17	4×10^{-8}
MIA-by-Sex	*Il34*	3.14	5×10^{-8}
MIA-by-Sex	*Hspa2*	3.14	5×10^{-8}
MIA-by-Sex	*Dnajb13*	3.27	5×10^{-8}
MIA-by-Sex	*Loc100738720*	3.30	5×10^{-8}
MIA-by-Sex	*Sema7a*	3.14	6×10^{-8}
MIA-by-Sex	*Sept4*	3.12	6×10^{-8}
MIA-by-Sex	*F5*	-3.1	6×10^{-8}
MIA-by-Sex	*Ldlr*	3.11	7×10^{-8}
MIA-by-Sex	*Lrrc75b*	3.30	8×10^{-8}
MIA-by-Sex	*Kcna1*	3.11	8×10^{-8}
MIA-by-Sex	*Fcgr1a*	3.14	9×10^{-8}
MIA-by-Sex	*Mob3b*	3.11	1×10^{-8}
MIA-by-Sex	*Nr2f1*	3.09	1×10^{-8}
MIA-by-Sex	*Itgb4*	3.09	1×10^{-8}
MIA-by-Sex	*Galnt6*	3.11	1×10^{-8}
MIA-by-Sex	*Pla1a*	3.19	2×10^{-8}
MIA-by-Sex	*Anln*	3.04	2×10^{-8}
MIA-by-Sex	*Rtkn*	3.04	2×10^{-8}
MIA-by-Sex	*Agrp*	3.49	2×10^{-8}
MIA-by-Sex	*Sox8*	3.02	2×10^{-8}
MIA-by-Sex	*Sirt2*	3.01	2×10^{-8}
MIA-by-Sex	*Zeb2*	3.02	2×10^{-8}
MIA-by-Sex	*Adcyap1r1*	3.01	2×10^{-8}
MIA-by-Sex	*Loc100157711*	3.08	3×10^{-8}
MIA-by-Sex	*Lrrn4cl*	3.03	3×10^{-8}
MIA-by-Sex	*Marcksl1*	3.00	3×10^{-8}
MIA-by-Sex	*Loc102162293*	3.11	3×10^{-8}
MIA-by-Sex	*Cldn19*	3.72	3×10^{-8}

MIA-by-Sex	*Lrrc55*	3.01	3×10^{-8}
MIA-by-Sex	*Lrrc38*	3.11	3×10^{-8}
MIA-by-Sex	*Nrgn*	-2.94	4×10^{-8}
MIA-by-Sex	*Wif1*	3.07	4×10^{-8}
MIA-by-Sex	*Loc110257816*	3.05	5×10^{-8}
MIA-by-Sex	*Loc100623257*	3.11	5×10^{-8}
MIA-by-Sex	*Arsb*	2.94	6×10^{-8}
MIA-by-Sex	*Hmgcs1*	2.94	6×10^{-8}
MIA-by-Sex	*Cmya5*	2.98	6×10^{-8}
MIA-by-Sex	*Slc26a7*	2.96	6×10^{-8}
MIA-by-Sex	*Aatk*	2.91	7×10^{-8}
MIA-by-Sex	*Pygm*	2.91	8×10^{-8}
MIA-by-Sex	*Loc110261221*	2.91	8×10^{-8}
MIA-by-Sex	*Psmb9*	2.94	8×10^{-8}
MIA-by-Sex	*Gas2l2*	3.00	8×10^{-8}
MIA-by-Sex	*Plac9*	2.93	9×10^{-8}
MIA-by-Sex	*Unc13c*	-2.94	1×10^{-7}
MIA-by-Sex	*Otp*	3.36	1×10^{-7}
MIA-by-Sex	*Sfrp5*	2.97	1×10^{-7}
MIA-by-Sex	*Cabp1*	-2.87	1×10^{-7}
MIA-by-Sex	*Prr5l*	2.88	1×10^{-7}
MIA-by-Sex	*Ifit3*	2.87	1×10^{-7}
MIA-by-Sex	*Nlrc5*	2.87	1×10^{-7}
MIA-by-Sex	*Clec18c*	2.85	2×10^{-7}
MIA-by-Sex	*Inf2*	2.85	2×10^{-7}
MIA-by-Sex	*Fscn1*	2.84	2×10^{-7}
MIA-by-Sex	*Pmp22*	2.84	2×10^{-7}
MIA-by-Sex	*Ada2*	2.85	2×10^{-7}
MIA-by-Sex	*Loc110257199*	2.96	2×10^{-7}
MIA-by-Sex	*Ltc4s*	2.96	2×10^{-7}
MIA-by-Sex	*Plcd3*	2.82	2×10^{-7}
MIA-by-Sex	*Tac1*	2.87	2×10^{-7}
MIA-by-Sex	*Bcas1*	2.81	2×10^{-7}
MIA-by-Sex	*Ngb*	2.92	2×10^{-7}
MIA-by-Sex	*Notum*	2.88	3×10^{-7}
MIA-by-Sex	*Fndc9*	2.96	3×10^{-7}
MIA-by-Sex	*Prph*	2.87	3×10^{-7}
MIA-by-Sex	*Nwd1*	2.86	3×10^{-7}
MIA-by-Sex	*Ambn*	2.93	3×10^{-7}
MIA-by-Sex	*Rarres2*	2.78	3×10^{-7}

MIA-by-Sex	*Kcnk13*	2.91	3×10^{-7}
MIA-by-Sex	*Isg20*	3.38	4×10^{-7}
MIA-by-Sex	*Cfb*	2.80	4×10^{-7}
MIA-by-Sex	*Loc100155195*	2.77	4×10^{-7}
MIA-by-Sex	*Tmem151a*	2.74	5×10^{-7}
MIA-by-Sex	*Ccl8*	2.9	5×10^{-7}
MIA-by-Sex	*Loc100524923*	2.77	6×10^{-7}
MIA-by-Sex	*Adcy1*	-2.69	7×10^{-7}
MIA-by-Sex	*Cthrc1*	2.76	7×10^{-7}
MIA-by-Sex	*Flvcr2*	2.78	7×10^{-7}
MIA-by-Sex	*Atoh8*	2.75	1×10^{-6}
MIA-by-Sex	*Cecr6*	-2.7	1×10^{-6}
MIA-by-Sex	*Pltp*	2.68	1×10^{-6}
MIA-by-Sex	*Frmd1*	2.71	1×10^{-6}
MIA-by-Sex	*Loc100158003*	2.84	1×10^{-6}
MIA-by-Sex	*Cbln4*	2.95	1×10^{-6}
MIA-by-Sex	*Odf3b*	2.91	1×10^{-6}
MIA-by-Sex	*Ret*	2.71	1×10^{-6}
MIA-by-Sex	*Tmem132e*	2.76	1×10^{-6}
MIA-by-Sex	*Foxj1*	2.67	2×10^{-6}
MIA-by-Sex	*Slc13a3*	2.69	2×10^{-6}
MIA-by-Sex	*Scrt1*	-2.62	2×10^{-6}
MIA-by-Sex	*St8sia5*	2.61	2×10^{-6}
MIA-by-Sex	*Col5a3*	2.62	2×10^{-6}
MIA-by-Sex	*Ube2l6*	2.62	2×10^{-6}
MIA-by-Sex	*Pm20d1*	2.65	2×10^{-6}
MIA-by-Sex	*Agpat4*	2.6	3×10^{-6}
MIA-by-Sex	*Znf469*	2.66	3×10^{-6}
MIA-by-Sex	*Vcam1*	2.60	3×10^{-6}
MIA-by-Sex	*Ttr*	2.83	3×10^{-6}
MIA-by-Sex	*Tmem155*	-2.74	3×10^{-6}
MIA-by-Sex	*Draxin*	2.69	3×10^{-6}
MIA-by-Sex	*Foxc2*	2.69	3×10^{-6}
MIA-by-Sex	*Bst2*	2.59	3×10^{-6}
MIA-by-Sex	*Casp1*	2.64	3×10^{-6}
MIA-by-Sex	*Trem2*	2.63	3×10^{-6}
MIA-by-Sex	*Wnt2b*	2.65	3×10^{-6}
MIA-by-Sex	*Grid1*	2.63	4×10^{-6}
MIA-by-Sex	*Loc110257701*	2.77	4×10^{-6}
MIA-by-Sex	*Mfap5*	2.75	4×10^{-6}

MIA-by-Sex	*Dapk2*	2.60	4×10^{-6}
MIA-by-Sex	*Sept8*	2.56	4×10^{-6}
MIA-by-Sex	*Oca2*	2.60	4×10^{-6}
MIA-by-Sex	*Loc100156694*	2.79	4×10^{-6}
MIA-by-Sex	*Elovl1*	2.55	4×10^{-6}
MIA-by-Sex	*Kif1c*	2.54	5×10^{-6}
MIA-by-Sex	*Loc100523672*	2.55	5×10^{-6}
MIA-by-Sex	*Mapk15*	2.54	5×10^{-6}
MIA-by-Sex	*Rasal3*	2.57	5×10^{-6}
MIA-by-Sex	*Ifit1*	2.54	5×10^{-6}
MIA-by-Sex	*Pip4k2a*	2.53	5×10^{-6}
MIA-by-Sex	*Cdhr1*	2.53	5×10^{-6}
MIA-by-Sex	*Cx3cr1*	2.54	5×10^{-6}
MIA-by-Sex	*Loc100620470*	-2.54	6×10^{-6}
MIA-by-Sex	*Gpr84*	2.78	6×10^{-6}
MIA-by-Sex	*Clcn2*	2.52	6×10^{-6}
MIA-by-Sex	*Ankrd65*	2.53	7×10^{-6}
MIA-by-Sex	*Lgi3*	2.51	7×10^{-6}
MIA-by-Sex	*Nrxn2*	2.50	7×10^{-6}
MIA-by-Sex	*Adora3*	2.52	7×10^{-6}
MIA-by-Sex	*Cd2*	2.60	9×10^{-6}
MIA-by-Sex	*Csf3r*	2.64	2×10^{-5}
MIA-by-Sex	*Irx6*	2.50	2×10^{-5}
MIA-by-Sex	*F2*	2.54	2×10^{-5}
MIA-by-Sex	*Lpar2*	2.52	2×10^{-5}
MIA-by-Sex	*Hdc*	2.57	2×10^{-5}
MIA-by-Sex	*Loc110259224*	2.7	2×10^{-5}
MIA-by-Sex	*Npw*	2.63	3×10^{-5}
MIA-by-Sex	*Cbln1*	2.78	3×10^{-5}
MIA-by-Sex	*Loc106505443*	2.74	3×10^{-4}
Weaning	*Sst*	-4.55	2×10^{-8}
Weaning	*Camkv*	-4.25	7×10^{-8}
Weaning	*Loc102161214*	-5.20	3×10^{-8}
Weaning	*Avp*	3.81	2×10^{-8}
Weaning	*Gabrq*	-3.93	9×10^{-8}
Weaning	*Vsnl1*	-3.62	3×10^{-8}
Weaning	*Draxin*	-3.63	5×10^{-8}
Weaning	*Loc100511183*	-3.52	6×10^{-8}
Weaning	*Cpne6*	-3.33	4×10^{-8}
Weaning	*Sult1a3*	-3.24	1×10^{-8}

Weaning	*Slc5a5*	-3.19	6×10^{-8}
Weaning	*Gal*	-3.11	3×10^{-8}
Weaning	*Nptx1*	-3.11	3×10^{-8}
Weaning	*Grin1*	-3.00	1×10^{-8}
Weaning	*Fndc9*	-3.07	1×10^{-8}
Weaning	*Calb2*	-2.97	2×10^{-8}
Weaning	*Loc100158003*	-3.10	2×10^{-8}
Weaning	*Loc110256043*	-2.86	2×10^{-8}
Weaning	*Npy*	-2.90	4×10^{-8}
Weaning	*Cacna2d3*	-2.86	5×10^{-8}
Weaning	*Gfra3*	-2.78	9×10^{-8}
Weaning	*Atp5pd*	-2.77	1×10^{-8}
Weaning	*Nxph1*	-2.85	2×10^{-8}
Weaning	*Prss56*	-2.74	2×10^{-8}
Weaning	*Gabra3*	-2.72	3×10^{-8}
Weaning	*Odf3b*	-2.89	5×10^{-8}
Weaning	*Slc6a7*	-2.71	5×10^{-8}
Weaning	*Entpd2*	-2.71	5×10^{-8}
Weaning	*Osr1*	-2.73	5×10^{-8}
Weaning	*Loc110257903*	-3.28	6×10^{-8}
Weaning	*Notum*	-2.65	3×10^{-8}
Weaning	*Tmem196*	-2.73	7×10^{-8}
Weaning	*Spata18*	-2.61	7×10^{-8}
Weaning	*Cpxm2*	-2.52	8×10^{-8}
Weaning	*Gpr50*	-2.73	1×10^{-8}
Weaning	*Cbln4*	-2.58	2×10^{-8}
Weaning	*Rgs14*	-2.56	2×10^{-8}
Weaning	*Hdc*	-2.57	3×10^{-8}
Weaning	*Gjb2*	-2.55	6×10^{-8}
Weaning	*Loc100525452*	-2.50	1×10^{-8}
Weaning	*Ubd*	-2.60	2×10^{-7}
Weaning	*Eif1ay*	4.88	3×10^{-6}
Weaning	*Loc100624590*	3.64	3×10^{-4}
Weaning	*Loc110257905*	3.57	5×10^{-4}
Weaning	*Loc100624149*	2.53	9×10^{-4}
Weaning	*Loc110255320*	3.18	4×10^{-2}
MIA	*Loc396781*	-7.49	6×10^{-8}
MIA	*Gabrq*	6.64	5×10^{-8}
MIA	*Loc100038329*	-5.61	9×10^{-8}
MIA	*Slc17a7*	-5.15	3×10^{-8}

MIA	*Loc100125542*	-4.52	2×10^{-8}
MIA	*Loc100152327*	-4.40	3×10^{-8}
MIA	*Loc110258704*	-5.01	4×10^{-8}
MIA	*Slc5a5*	4.68	4×10^{-8}
MIA	*C14h10orf105*	4.44	3×10^{-8}
MIA	*Loc102167964*	-4.28	7×10^{-8}
MIA	*Gal*	4.17	8×10^{-8}
MIA	*Ecel1*	4.04	3×10^{-8}
MIA	*Marco*	-4.42	5×10^{-8}
MIA	*Rasgrp1*	-3.76	1×10^{-8}
MIA	*Neurod2*	-3.76	4×10^{-8}
MIA	*Agrp*	4.18	7×10^{-8}
MIA	*Foxg1*	-3.70	2×10^{-8}
MIA	*Nkx6-1*	3.90	3×10^{-8}
MIA	*Loc110258326*	-4.39	3×10^{-8}
MIA	*Loc110259983*	-3.92	2×10^{-8}
MIA	*Agt*	3.53	5×10^{-8}
MIA	*Ddn*	-3.36	6×10^{-8}
MIA	*Klrk1*	-3.69	2×10^{-8}
MIA	*Pdyn*	3.36	2×10^{-8}
MIA	*F5*	-3.19	2×10^{-8}
MIA	*Calb2*	3.32	5×10^{-8}
MIA	*Sult1a3*	3.29	1×10^{-8}
MIA	*Col2a1*	3.24	2×10^{-8}
MIA	*Gfra3*	3.26	2×10^{-8}
MIA	*Grin2a*	-3.07	6×10^{-8}
MIA	*Unc13c*	-3.03	9×10^{-8}
MIA	*Gfap*	3.13	1×10^{-8}
MIA	*Comp*	3.38	1×10^{-8}
MIA	*Cd3e*	-3.38	1×10^{-8}
MIA	*Slc22a2*	3.34	1×10^{-8}
MIA	*Mpped1*	-2.98	2×10^{-8}
MIA	*Camk2a*	-2.95	3×10^{-8}
MIA	*Tmem155*	-3.09	4×10^{-8}
MIA	*Cabp1*	-2.85	3×10^{-8}
MIA	*Cd6*	-3.68	6×10^{-8}
MIA	*Cd3g*	-3.38	6×10^{-8}
MIA	*Entpd2*	2.95	8×10^{-8}
MIA	*Loc100623670*	-3.38	8×10^{-8}
MIA	*Zic1*	2.89	1×10^{-8}

MIA	*Rflnb*	2.85	2×10^{-8}
MIA	*Tac1*	2.87	2×10^{-8}
MIA	*Cbln1*	2.91	3×10^{-8}
MIA	*Osr1*	2.89	5×10^{-8}
MIA	*Kctd4*	2.89	5×10^{-8}
MIA	*Lck*	-2.92	5×10^{-8}
MIA	*Prss56*	2.82	6×10^{-8}
MIA	*Nrgn*	-2.65	1×10^{-8}
MIA	*Cd3d*	-3.29	1×10^{-8}
MIA	*Itk*	-3.21	1×10^{-8}
MIA	*Tpo*	2.79	1×10^{-8}
MIA	*Adgre1*	-3.28	1×10^{-8}
MIA	*Hapln2*	2.73	2×10^{-8}
MIA	*Loc100620470*	-2.63	2×10^{-8}
MIA	*Adcy1*	-2.59	3×10^{-8}
MIA	*Cxcr6*	-3.62	3×10^{-8}
MIA	*Fndc9*	2.74	3×10^{-8}
MIA	*Loc110257701*	2.87	5×10^{-8}
MIA	*Loc110261683*	2.68	5×10^{-8}
MIA	*Ccl5*	-2.60	7×10^{-8}
MIA	*Sfrp5*	2.70	9×10^{-8}
MIA	*Slc1a2*	-2.52	9×10^{-8}
MIA	*Lhx5*	2.76	1×10^{-8}
MIA	*Loc110257816*	2.69	1×10^{-8}
MIA	*Loc100621559*	-2.85	1×10^{-8}
MIA	*Klrd1*	-2.85	2×10^{-8}
MIA	*Cpxm2*	2.58	2×10^{-8}
MIA	*Fezf1*	2.61	3×10^{-8}
MIA	*Gzma*	-2.86	3×10^{-8}
MIA	*Cd8b*	-3.27	3×10^{-8}
MIA	*Lrrn4cl*	2.57	3×10^{-8}
MIA	*Gpr50*	2.83	4×10^{-8}
MIA	*Hdc*	2.67	4×10^{-8}
MIA	*Loc100038328*	-3.54	5×10^{-8}
MIA	*Cnmd*	2.55	5×10^{-8}
MIA	*T*	2.78	7×10^{-8}
MIA	*Slc18a2*	2.54	1×10^{-8}
MIA	*Loc106505443*	3.25	1×10^{-8}
MIA	*Loc100519314*	-2.65	2×10^{-8}
MIA	*Sit1*	-2.78	2×10^{-8}

MIA	*Prf1*	-2.74	3×10^{-8}
MIA	*Odf3b*	2.56	3×10^{-8}
MIA	*Eomes*	-2.61	3×10^{-8}
MIA	*Gzmk*	-2.66	6×10^{-8}
MIA	*Cd244*	-2.74	2×10^{-8}
MIA	*Npw*	2.55	4×10^{-8}
MIA	*Loc100620198*	-2.55	6×10^{-8}
MIA	*Loc110258827*	-2.97	1×10^{-7}
MIA	*Msmp*	2.89	3×10^{-7}
MIA	*Loc100518860*	-2.73	3×10^{-6}
MIA	*Loc110258673*	-2.68	3×10^{-6}
Sex	*Loc100624149*	12.14	3×10^{-8}
Sex	*Loc100624590*	13.44	7×10^{-8}
Sex	*Loc110257905*	13.17	7×10^{-8}
Sex	*Eif1ay*	13.91	1×10^{-8}
Sex	*Loc100625207*	10.32	6×10^{-8}
Sex	*Loc110255320*	12.15	1×10^{-8}
Sex	*Loc110257883*	12.15	1×10^{-8}
Sex	*Hapln2*	-8.55	5×10^{-8}
Sex	*Loc110255257*	10.53	1×10^{-8}
Sex	*Gjb1*	-8.77	3×10^{-8}
Sex	*Mobp*	-7.91	3×10^{-8}
Sex	*Mbp*	-7.90	3×10^{-8}
Sex	*Plp1*	-7.80	5×10^{-8}
Sex	*Ermn*	-7.74	2×10^{-8}
Sex	*Gfap*	-7.24	2×10^{-8}
Sex	*Mog*	-7.39	2×10^{-8}
Sex	*Sst*	-7.49	7×10^{-8}
Sex	*Mal*	-7.04	4×10^{-8}
Sex	*Loc110257896*	9.18	1×10^{-8}
Sex	*Mag*	-6.61	1×10^{-8}
Sex	*Ugt8*	-6.57	3×10^{-8}
Sex	*Mlc1*	-6.44	2×10^{-8}
Sex	*Gjc2*	-6.43	3×10^{-8}
Sex	*Loc110261683*	-6.58	5×10^{-8}
Sex	*Fa2h*	-6.32	6×10^{-8}
Sex	*Tf*	-6.24	1×10^{-8}
Sex	*Slc14a1*	-6.45	6×10^{-8}
Sex	*Loc110257894*	6.66	3×10^{-8}
Sex	*Grin1*	-6.04	1×10^{-8}

Sex	*Camkv*	-6.10	3×10^{-8}
Sex	*Grm3*	-6.43	6×10^{-8}
Sex	*Cmtm5*	-6.06	1×10^{-8}
Sex	*Sgk2*	-5.87	4×10^{-8}
Sex	*Loc100624329*	5.73	3×10^{-8}
Sex	*Gpr37l1*	-5.52	3×10^{-8}
Sex	*Pou3f3*	-5.65	2×10^{-8}
Sex	*Nkx6-2*	-5.60	4×10^{-8}
Sex	*Tubb4a*	-5.37	1×10^{-8}
Sex	*Ttyh1*	-5.40	2×10^{-8}
Sex	*Calb2*	-5.40	4×10^{-8}
Sex	*Atp10b*	-5.42	8×10^{-8}
Sex	*Cpne6*	-5.36	9×10^{-8}
Sex	*Tmem88b*	-5.32	1×10^{-8}
Sex	*Olig1*	-5.17	2×10^{-8}
Sex	*Sox10*	-5.13	9×10^{-8}
Sex	*Sh3tc2*	-5.24	4×10^{-8}
Sex	*Fabp7*	-5.07	5×10^{-8}
Sex	*Adamts4*	-5.05	2×10^{-8}
Sex	*Sox21*	-5.33	6×10^{-8}
Sex	*Opalin*	-4.96	6×10^{-8}
Sex	*Elfn2*	-4.97	8×10^{-8}
Sex	*Col11a2*	-4.96	3×10^{-8}
Sex	*Cbln1*	-5.22	4×10^{-8}
Sex	*Loc396706*	8.67	8×10^{-8}
Sex	*Bcan*	-4.84	1×10^{-8}
Sex	*Slc1a3*	-4.81	2×10^{-8}
Sex	*Slc6a7*	-4.90	4×10^{-8}
Sex	*Nkx6-1*	-4.97	4×10^{-8}
Sex	*Gabrq*	-4.92	2×10^{-8}
Sex	*Pdyn*	-4.75	2×10^{-8}
Sex	*Plxnb3*	-4.73	3×10^{-8}
Sex	*Kcnj10*	-4.69	5×10^{-8}
Sex	*Zic5*	-5.00	6×10^{-8}
Sex	*Ndp*	-4.78	2×10^{-8}
Sex	*Olig2*	-4.65	3×10^{-8}
Sex	*Npy*	-4.78	4×10^{-8}
Sex	*Evi2a*	-4.66	8×10^{-8}
Sex	*Synpo2l*	-4.67	1×10^{-8}
Sex	*Zic1*	-4.64	1×10^{-8}

Sex	*Snx22*	-4.57	1×10^{-8}
Sex	*Rab7b*	-4.64	2×10^{-8}
Sex	*Th*	-4.94	3×10^{-8}
Sex	*Fam135b*	-4.63	4×10^{-8}
Sex	*Agt*	-4.48	6×10^{-8}
Sex	*Efhd1*	-4.48	1×10^{-8}
Sex	*Gpr17*	-4.42	4×10^{-8}
Sex	*Paqr6*	-4.41	4×10^{-8}
Sex	*C14h10orf90*	-4.48	3×10^{-8}
Sex	*C14h10orf105*	-4.37	7×10^{-8}
Sex	*Kcnip1*	-4.43	7×10^{-8}
Sex	*Kctd4*	-4.52	1×10^{-8}
Sex	*Nptx1*	-4.37	1×10^{-8}
Sex	*Zdhhc22*	-4.44	2×10^{-8}
Sex	*Ppp1r14a*	-4.24	3×10^{-8}
Sex	*Gal*	-4.29	4×10^{-8}
Sex	*Igsf21*	-4.50	4×10^{-8}
Sex	*Gad1*	-4.27	6×10^{-8}
Sex	*Alox15*	4.19	9×10^{-8}
Sex	*Cbln4*	-4.39	4×10^{-8}
Sex	*Comp*	-4.53	8×10^{-8}
Sex	*Slc6a1*	-4.12	1×10^{-8}
Sex	*Slc5a11*	-4.19	1×10^{-8}
Sex	*Grid1*	-4.17	1×10^{-8}
Sex	*Adap1*	-4.08	2×10^{-8}
Sex	*Ache*	-4.05	5×10^{-8}
Sex	*Shisa9*	-4.15	5×10^{-8}
Sex	*Vsnl1*	-4.04	6×10^{-8}
Sex	*Ca14*	-4.13	7×10^{-8}
Sex	*Fndc9*	-4.12	9×10^{-8}
Sex	*Loc110255851*	-4.12	3×10^{-8}
Sex	*Irx1*	-4.34	5×10^{-8}
Sex	*Flt3*	-4.11	6×10^{-8}
Sex	*Slc16a4*	-4.10	6×10^{-8}
Sex	*Aqp4*	-3.89	1×10^{-8}
Sex	*Acan*	-4.17	5×10^{-8}
Sex	*S1pr5*	-3.88	7×10^{-8}
Sex	*Slc1a6*	-3.88	2×10^{-8}
Sex	*Loc100511183*	-3.85	2×10^{-8}
Sex	*Apod*	-3.76	2×10^{-8}

Sex	*Cldn11*	-3.75	3×10^{-8}
Sex	*C4h8orf46*	-3.75	6×10^{-8}
Sex	*Loc106510131*	-3.74	6×10^{-8}
Sex	*Slc4a10*	-3.82	7×10^{-8}
Sex	*Wnt7b*	-3.80	9×10^{-8}
Sex	*Nkd1*	-3.72	1×10^{-8}
Sex	*Apoe*	-3.66	3×10^{-8}
Sex	*Gal3st1*	-3.66	5×10^{-8}
Sex	*Loc110257910*	7.31	6×10^{-8}
Sex	*Pdlim4*	-3.67	2×10^{-8}
Sex	*Pllp*	-3.58	2×10^{-8}
Sex	*Draxin*	-3.65	5×10^{-8}
Sex	*Agrp*	-3.70	5×10^{-8}
Sex	*Zic2*	-3.58	6×10^{-8}
Sex	*Cldn10*	-3.56	2×10^{-8}
Sex	*Myrf*	-3.47	2×10^{-8}
Sex	*Mgat5b*	-3.57	4×10^{-8}
Sex	*Tmem132e*	-3.53	8×10^{-8}
Sex	*Kcna1*	-3.42	1×10^{-8}
Sex	*Hepacam*	-3.39	1×10^{-8}
Sex	*Cldn19*	-4.25	2×10^{-8}
Sex	*Sdcbp2*	-3.47	2×10^{-8}
Sex	*Loc110256043*	-3.40	3×10^{-8}
Sex	*Otp*	-3.73	4×10^{-8}
Sex	*Fez1*	-3.34	5×10^{-8}
Sex	*Gdf1*	-3.33	5×10^{-8}
Sex	*Smoc1*	-3.34	6×10^{-8}
Sex	*Rab33a*	-3.38	6×10^{-8}
Sex	*Slc1a2*	-3.31	8×10^{-8}
Sex	*Fam131b*	-3.34	8×10^{-8}
Sex	*Cacna2d3*	-3.38	1×10^{-8}
Sex	*Neu4*	-3.33	1×10^{-8}
Sex	*Rasgrf1*	-3.35	1×10^{-8}
Sex	*Lcat*	-3.41	1×10^{-8}
Sex	*C1ql3*	-3.33	2×10^{-8}
Sex	*Fndc10*	-3.26	8×10^{-8}
Sex	*Slc5a5*	-3.25	1×10^{-8}
Sex	*Nxph1*	-3.29	1×10^{-8}
Sex	*Cnp*	-3.17	2×10^{-8}
Sex	*Arhgdig*	-3.21	2×10^{-8}

Sex	*Notum*	-3.24	2×10^{-8}
Sex	*Dthd1*	-3.29	3×10^{-8}
Sex	*Osr1*	-3.22	4×10^{-8}
Sex	*Rgs14*	-3.22	4×10^{-8}
Sex	*Fabp3*	-3.14	6×10^{-8}
Sex	*B4galnt1*	-3.13	7×10^{-8}
Sex	*Omg*	-3.11	8×10^{-8}
Sex	*Atp13a4*	-3.19	8×10^{-8}
Sex	*Pla2r1*	-3.12	8×10^{-8}
Sex	*Chn2*	-3.11	1×10^{-8}
Sex	*Lgi4*	-3.05	3×10^{-8}
Sex	*Glra2*	-3.09	5×10^{-8}
Sex	*Stmn4*	-2.99	9×10^{-8}
Sex	*Ada*	-3.03	9×10^{-8}
Sex	*Csmd1*	-3.01	1×10^{-8}
Sex	*Cers1*	-2.95	2×10^{-8}
Sex	*Sphk1*	2.93	3×10^{-8}
Sex	*Rarres1*	-2.91	4×10^{-8}
Sex	*Loc110257598*	-2.99	4×10^{-8}
Sex	*Gpr37*	-2.91	5×10^{-8}
Sex	*Map6d1*	-2.91	6×10^{-8}
Sex	*Loc110257903*	-3.59	6×10^{-8}
Sex	*Asic2*	-2.92	7×10^{-8}
Sex	*Sult1a3*	-2.91	7×10^{-8}
Sex	*Homer3*	-2.89	7×10^{-8}
Sex	*Aspa*	-2.99	8×10^{-8}
Sex	*Ngb*	-2.92	2×10^{-8}
Sex	*Loc100524517*	-2.97	2×10^{-8}
Sex	*Tac1*	-2.86	2×10^{-8}
Sex	*Emilin2*	-2.85	3×10^{-8}
Sex	*Shmt1*	-2.84	3×10^{-8}
Sex	*Pla1a*	-2.98	3×10^{-8}
Sex	*Phyhip*	-2.84	3×10^{-8}
Sex	*Ncan*	-2.82	3×10^{-8}
Sex	*Slitrk3*	-2.95	4×10^{-8}
Sex	*Hs6st3*	-2.86	6×10^{-8}
Sex	*Gabra3*	-2.80	6×10^{-8}
Sex	*Lrrc71*	-2.84	9×10^{-8}
Sex	*Erbb3*	-2.77	9×10^{-8}
Sex	*Adcyap1r1*	-2.76	1×10^{-8}

Sex	*Cacng4*	-2.78	1×10^{-8}
Sex	*Apln*	-2.77	1×10^{-8}
Sex	*Nr2f1*	-2.75	1×10^{-8}
Sex	*Prima1*	-2.77	1×10^{-8}
Sex	*Cpxm2*	-2.75	1×10^{-8}
Sex	*Cp*	-2.78	1×10^{-8}
Sex	*S100a1*	-2.73	2×10^{-8}
Sex	*Ido1*	-2.90	2×10^{-8}
Sex	*Cnmd*	-2.75	2×10^{-8}
Sex	*Ltc4s*	-2.84	3×10^{-8}
Sex	*Tmem196*	-2.85	4×10^{-8}
Sex	*Entpd2*	-2.72	4×10^{-8}
Sex	*Sema7a*	-2.69	5×10^{-8}
Sex	*Zeb2*	-2.68	5×10^{-8}
Sex	*Prr18*	-2.67	7×10^{-8}
Sex	*Flvcr2*	-2.70	2×10^{-8}
Sex	*Gjb6*	-2.67	2×10^{-8}
Sex	*Acsbg1*	-2.60	3×10^{-8}
Sex	*Fgfr3*	-2.60	3×10^{-8}
Sex	*Kcna6*	-2.59	3×10^{-8}
Sex	*Gjb2*	-2.71	4×10^{-8}
Sex	*Daam2*	-2.57	5×10^{-8}
Sex	*Dact2*	-2.73	5×10^{-8}
Sex	*Fgfr4*	-2.65	5×10^{-8}
Sex	*Tmem144*	-2.57	5×10^{-8}
Sex	*Ramp3*	-2.56	6×10^{-8}
Sex	*Crsp-2*	-2.59	6×10^{-8}
Sex	*Loc100157711*	-2.63	8×10^{-8}
Sex	*Tbx1*	-2.71	1×10^{-8}
Sex	*Ecel1*	-2.52	1×10^{-8}
Sex	*Fam159b*	-2.60	1×10^{-8}
Sex	*Sirt2*	-2.52	1×10^{-8}
Sex	*Cfb*	-2.52	2×10^{-8}
Sex	*Nrxn3*	-2.54	2×10^{-8}
Sex	*Dnajb13*	-2.56	3×10^{-8}
Sex	*Loc100524923*	-2.52	3×10^{-8}
Sex	*Loc110255218*	-2.55	3×10^{-8}
Sex	*Loc100512593*	-2.56	3×10^{-8}
Sex	*Gabra4*	-2.50	4×10^{-8}
Sex	*Gtsf1*	2.63	5×10^{-8}

Sex	*Loc110257199*	-2.58	6×10^{-8}
Sex	*Otos*	-2.52	7×10^{-8}

[1] LogFC > 0 (LogFC < 0) denotes (for main and interaction effects) gene over-expression (under-expression) in control relative to MIA-exposed pigs, pigs exposed to weaning stress relative to not stressed (nursed), and males relative to females.

Supplemental Table S2. Gene Ontology biological processes and Kyoto Encyclopedia of Genes and Genomes pathways enriched (normalized enrichment score NES or ratio > 1.6, False Discovery Rate (FDR) adjusted *p*-value < 0.05) identified by Gene Set Enrichment Analysis (GSEA) and by Over-representation Analysis (ORA using genes with FDR *p*-value < 0.05) for the interactions and main effects of maternal immune activation (MIA), weaning, and sex in pituitary gland.

Gene Set Enrichment Analysis (GSEA)

		Sex		Weaning		MIA		MIA x Sex		Weaning x Sex		Weaning x MIA	
Category		NES	FDR[3]	NES	FDR	NES	FDR	NES	FDR	NES	FDR	NES	FDR
GO Biological Process[1]													
GO:0007272	ensheathment of neurons	-1.8	2×10^{-8}			1.6	4×10^{-2}	1.8	5×10^{-3}			-2.2	2×10^{-8}
GO:0031099	regeneration	-1.7	1×10^{-2}			1.6	4×10^{-2}	1.7	3×10^{-2}				
GO:0007059	chromosome segregation			-2.0	1×10^{-3}								
GO:0007218	neuropeptide signaling pathway			-2.0	3×10^{-3}	1.8	2×10^{-2}			2.0	5×10^{-3}	1.8	2×10^{-2}
GO:0048285	organelle fission			-2.0	3×10^{-3}								
GO:0050803	regulation of synapse structure or activity			-1.8	2×10^{-2}								
GO:0043062	extracellular structure organization					2.0	2×10^{-8}	1.7	3×10^{-2}				
GO:0001503	ossification					2.0	5×10^{-3}						
GO:0042303	molting cycle					1.8	1×10^{-2}			-2.0	8×10^{-3}	2.0	2×10^{-2}
GO:0060485	mesenchyme development					1.8	1×10^{-2}						
GO:0061458	reproductive system development					1.8	2×10^{-2}					1.7	4×10^{-2}
GO:0008544	epidermis development					1.7	2×10^{-2}					2.0	3×10^{-2}
GO:0043588	skin development					1.8	2×10^{-2}					2.0	2×10^{-2}
GO:0001501	skeletal system development					1.7	2×10^{-2}						
GO:0055123	digestive system development					1.7	2×10^{-2}						
GO:0001763	morphogenesis of a branching structure					1.8	2×10^{-2}			-2.0	1×10^{-2}		
GO:0001655	urogenital system development					1.7	3×10^{-2}						
GO:0007389	pattern specification process					1.7	3×10^{-2}					2.0	2×10^{-2}
GO:0048880	sensory system development					1.7	4×10^{-2}						

GO:0048736	appendage development					1.6	4×10^{-2}						
GO:0061448	connective tissue development					1.7	4×10^{-2}						
GO:0031214	biomineral tissue development					1.6	4×10^{-2}						
GO:0031016	pancreas development					1.6	4×10^{-2}						
GO:0046683	response to organophosphorus					1.6	4×10^{-2}					2.0	3×10^{-2}
GO:0060541	respiratory system development					1.6	5×10^{-2}					1.8	3×10^{-2}
GO:0044282	small molecule catabolic process					1.6	5×10^{-2}						
GO:0021675	nerve development							-1.8	3×10^{-2}				
GO:0048732	gland development									-2.0	3×10^{-2}		
GO:0070661	leukocyte proliferation											1.8	2×10^{-2}
GO:0007492	endoderm development											1.8	2×10^{-2}
GO:0072376	protein activation cascade											1.8	3×10^{-2}
GO:0019932	second-messenger-mediated signaling											1.7	5×10^{-2}
KEGG Pathways[2]													
ssc05033	Nicotine addiction	-1.8	9×10^{-3}	-2.1	2×10^{-8}					2.0	3×10^{-3}		
ssc05031	Amphetamine addiction	-1.6	5×10^{-2}										
ssc05032	Morphine addiction			-1.8	5×10^{-3}					1.7	3×10^{-2}		
ssc04727	GABAergic synapse			-1.8	5×10^{-3}					1.7	3×10^{-2}		
ssc04110	Cell cycle			-1.8	7×10^{-3}								
ssc03030	DNA replication			-1.8	7×10^{-3}								
ssc04114	Oocyte meiosis			-1.8	1×10^{-2}								
ssc04724	Glutamatergic synapse			-1.8	1×10^{-2}								
ssc04971	Gastric acid secretion			-1.8	1×10^{-2}								
ssc00190	Oxidative phosphorylation			-1.8	1×10^{-2}					2.0	2×10^{-3}	-2.0	2×10^{-2}
ssc00534	Glycosaminoglycan biosynthesis			-1.7	1×10^{-2}								
ssc03460	Fanconi anemia pathway			-1.7	1×10^{-2}								
ssc04974	Protein digestion and absorption			-1.7	2×10^{-2}	2.0	2×10^{-3}					2.0	2×10^{-3}
ssc04540	Gap junction			-1.7	2×10^{-2}								
ssc00500	Starch and sucrose metabolism			2.0	2×10^{-2}								
ssc03010	Ribosome			2.0	3×10^{-2}	-2.2	2×10^{-8}			2.0	2×10^{-8}	-2.3	2×10^{-8}
ssc03440	Homologous recombination			-1.7	4×10^{-2}								
ssc04720	Long-term potentiation			-1.7	4×10^{-2}	-1.7	4×10^{-2}					-1.8	3×10^{-2}
ssc04660	T cell receptor signaling pathway					-2.2	2×10^{-8}						
ssc04940	Type I diabetes mellitus					-2.0	2×10^{-4}					1.7	3×10^{-2}
ssc04650	Natural killer cell mediated cytotoxicity					-2.1	2×10^{-4}						
ssc05330	Allograft rejection					-2.1	3×10^{-4}					2.1	1×10^{-4}
ssc05340	Primary immunodeficiency					-2.1	3×10^{-4}						
ssc05332	Graft-versus-host disease					-2.0	9×10^{-4}					2.3	2×10^{-8}

ssc05162	Measles					-1.8	2×10^{-2}					1.7	2×10^{-2}
ssc05416	Viral myocarditis					-1.8	2×10^{-2}					2.1	1×10^{-4}
ssc04640	Hematopoietic cell lineage					-1.8	2×10^{-2}					2.0	4×10^{-3}
ssc05320	Autoimmune thyroid disease					-1.8	2×10^{-2}					2.1	1×10^{-4}
ssc04612	Antigen processing and presentation					-1.8	2×10^{-2}					2.4	2×10^{-8}
ssc04659	Th17 cell differentiation					-1.7	3×10^{-2}					2.0	3×10^{-3}
ssc04721	Synaptic vesicle cycle					-1.7	3×10^{-2}			1.8	2×10^{-2}		
ssc04672	Intestinal immune network for IgA production					-1.7	4×10^{-2}					1.8	2×10^{-2}
ssc04550	Signaling pathways regulating pluripotency of stem cells					1.8	5×10^{-2}						
ssc00340	Histidine metabolism					1.8	5×10^{-2}						
ssc04658	Th1 and Th2 cell differentiation					-1.7	5×10^{-2}					2.0	3×10^{-3}
ssc05150	Staphylococcus aureus infection							1.8	1×10^{-2}			2.5	2×10^{-8}
ssc00100	Steroid biosynthesis							1.7	3×10^{-2}			-2.0	9×10^{-3}
ssc05321	Inflammatory bowel disease (IBD)											2.2	2×10^{-8}
ssc04610	Complement and coagulation cascades											2.2	2×10^{-8}
ssc05144	Malaria											2.2	2×10^{-8}
ssc05164	Influenza A											2.0	3×10^{-3}
ssc05310	Asthma											2.0	4×10^{-3}
ssc04621	NOD-like receptor signaling pathway											2.0	4×10^{-3}
ssc04512	ECM-receptor interaction											2.0	4×10^{-3}
ssc05133	Pertussis											2.0	4×10^{-3}
ssc04724	Glutamatergic synapse											-2.0	1×10^{-2}
ssc00900	Terpenoid backbone biosynthesis											-2.0	1×10^{-2}
ssc00670	One carbon pool by folate											-2.0	1×10^{-2}
ssc04911	Insulin secretion											-1.8	3×10^{-2}
ssc05323	Rheumatoid arthritis											1.7	3×10^{-2}
ssc04962	Vasopressin-regulated water reabsorption											-1.8	3×10^{-2}
ssc04725	Cholinergic synapse											-1.8	3×10^{-2}
ssc05322	Systemic lupus erythematosus											1.7	4×10^{-2}
ssc04620	Toll-like receptor signaling pathway											1.7	4×10^{-2}
ssc04933	AGE-RAGE signaling pathway in diabetic complications											1.7	4×10^{-2}

Over-Representation Analysis (ORA)

	Sex		Weaning		MIA		MIA x Sex		Weaning x Sex		Weaning x MIA	
Category	Ratio	FDR	Ratio	FDR	Ratio	FDR	Ratio	FDR	Ratio	FDR	Ratio	FDR

GO Biological Process[1]													
GO:0042391	regulation of membrane potential	4.3	7×10^{-6}										
GO:0048167	regulation of synaptic plasticity	5.6	5×10^{-4}										
GO:0016126	sterol biosynthetic process	7.6	1×10^{-3}					9.8	9×10^{-5}				
GO:0050890	cognition	4.4	2×10^{-3}										
GO:0050804	modulation of chemical synaptic transmission	3.5	2×10^{-3}										
GO:0099177	regulation of trans-synaptic signaling	3.5	2×10^{-3}										
GO:1901617	organic hydroxy compound biosynthetic process	3.8	2×10^{-3}					4.0	4×10^{-3}				
GO:0051480	regulation of cytosolic calcium ion concentration	3.4	2×10^{-3}			3.8	5×10^{-2}						
GO:0001505	regulation of neurotransmitter levels	3.3	3×10^{-3}					3.8	1×10^{-3}			5.0	1×10^{-2}
GO:0060078	regulation of postsynaptic membrane potential	6.1	3×10^{-3}										
GO:0016125	sterol metabolic process	4.6	6×10^{-3}					6.0	7×10^{-4}				
GO:0010001	glial cell differentiation	3.9	7×10^{-3}					4.0	2×10^{-2}			6.2	8×10^{-3}
GO:0048709	oligodendrocyte differentiation	6.2	8×10^{-3}					8.0	1×10^{-3}			12.5	2×10^{-3}
GO:0060041	retina development in camera-type eye	5.1	1×10^{-2}										
GO:0007611	learning or memory	4.1	1×10^{-2}										
GO:0061564	axon development	2.8	1×10^{-2}										
GO:0006937	regulation of muscle contraction	4.3	2×10^{-2}										
GO:0006694	steroid biosynthetic process	3.9	2×10^{-2}										
GO:0035637	multicellular organismal signaling	4.6	2×10^{-2}										
GO:0007187	G protein-coupled receptor signaling pathway, coupled to cyclic nucleotide second messenger	3.0	2×10^{-2}			4.1	3×10^{-2}						
GO:0007204	positive regulation of cytosolic calcium ion concentration	3.1	3×10^{-2}										
GO:0003013	circulatory system process	2.7	3×10^{-2}										
GO:0008203	cholesterol metabolic process	4.4	3×10^{-2}					5.7	4×10^{-3}				
GO:1902652	secondary alcohol metabolic process	4.4	3×10^{-2}					5.7	4×10^{-3}				
GO:0060291	long-term synaptic potentiation	6.6	3×10^{-2}										
GO:0006695	cholesterol biosynthetic process	6.6	3×10^{-2}					8.6	6×10^{-3}				
GO:1902653	secondary alcohol biosynthetic process	6.6	3×10^{-2}					8.6	6×10^{-3}				
GO:0010524	positive regulation of calcium ion transport into cytosol	6.6	3×10^{-2}					8.6	6×10^{-3}				
GO:0006836	neurotransmitter transport	3.2	3×10^{-2}					4.1	3×10^{-3}			5.1	2×10^{-2}
GO:0007628	adult walking behavior	8.0	3×10^{-2}										
GO:0019933	cAMP-mediated signaling	3.3	3×10^{-2}			5.1	2×10^{-2}	3.6	3×10^{-2}				

GO:0007189	adenylate cyclase-activating G protein-coupled receptor signaling pathway	3.5	3×10^{-2}			5.4	3×10^{-2}						
GO:0046456	icosanoid biosynthetic process	6.2	3×10^{-2}										
GO:0060079	excitatory postsynaptic potential	6.2	3×10^{-2}										
GO:0046165	alcohol biosynthetic process	3.8	3×10^{-2}										
GO:0007272	ensheathment of neurons	4.6	3×10^{-2}					6.7	2×10^{-3}			9.3	7×10^{-3}
GO:0008366	axon ensheathment	4.6	3×10^{-2}					6.7	2×10^{-3}			9.3	7×10^{-3}
GO:0008015	blood circulation	2.6	3×10^{-2}										
GO:0043547	positive regulation of GTPase activity	2.8	5×10^{-2}					3.4	3×10^{-2}				
GO:0030534	adult behavior	4.1	3×10^{-2}										
GO:0006942	regulation of striated muscle contraction	5.2	3×10^{-2}										
GO:0071805	potassium ion transmembrane transport	3.4	3×10^{-2}										
GO:1990573	potassium ion import across plasma membrane	7.4	3×10^{-2}										
GO:0008299	isoprenoid biosynthetic process	7.4	3×10^{-2}					9.5	1×10^{-2}				
GO:0071804	cellular potassium ion transport	3.4	3×10^{-2}										
GO:0050900	leukocyte migration	2.7	4×10^{-2}					4.6	3×10^{-5}				
GO:0007409	axonogenesis	2.7	4×10^{-2}										
GO:0008344	adult locomotory behavior	4.8	4×10^{-2}										
GO:0098656	anion transmembrane transport	3.2	5×10^{-2}									6.0	1×10^{-2}
GO:0051208	sequestering of calcium ion	4.3	5×10^{-2}					5.5	2×10^{-2}				
GO:0019935	cyclic-nucleotide-mediated signaling					5.1	2×10^{-2}						
GO:0043062	extracellular structure organization					4.8	2×10^{-2}	4.0	4×10^{-3}				
GO:0033077	T cell differentiation in thymus					9.8	2×10^{-2}						
GO:0007188	adenylate cyclase-modulating G protein-coupled receptor signaling pathway					4.6	2×10^{-2}						
GO:0006898	receptor-mediated endocytosis					4.7	3×10^{-2}						
GO:0006855	drug transmembrane transport					8.6	3×10^{-2}					12.5	2×10^{-3}
GO:0030199	collagen fibril organization					11.0	3×10^{-2}						
GO:0030198	extracellular matrix organization					5.0	3×10^{-2}	4.0	2×10^{-2}				
GO:0060326	cell chemotaxis							4.2	7×10^{-5}				
GO:0097529	myeloid leukocyte migration							5.8	9×10^{-5}				
GO:0071674	mononuclear cell migration							9.8	9×10^{-5}				
GO:0030595	leukocyte chemotaxis							4.8	2×10^{-4}				
GO:0002548	monocyte chemotaxis							11.5	5×10^{-4}				
GO:0071346	cellular response to interferon-gamma							6.9	1×10^{-3}				
GO:0002685	regulation of leukocyte migration							4.8	2×10^{-3}				

GO:0034341	response to interferon-gamma	6.1	3×10^{-3}		
GO:0071621	granulocyte chemotaxis	5.7	4×10^{-3}		
GO:0030593	neutrophil chemotaxis	6.5	4×10^{-3}		
GO:0042552	myelination	6.3	5×10^{-3}	8.5	2×10^{-2}
GO:0071675	regulation of mononuclear cell migration	9.2	5×10^{-3}		
GO:0002688	regulation of leukocyte chemotaxis	5.3	6×10^{-3}		
GO:0097530	granulocyte migration	5.3	6×10^{-3}		
GO:1990266	neutrophil migration	6.0	6×10^{-3}		
GO:0051928	positive regulation of calcium ion transport	6.0	6×10^{-3}		
GO:0006837	serotonin transport	15.3	7×10^{-3}		
GO:0006835	dicarboxylic acid transport	7.6	1×10^{-2}	12.0	2×10^{-2}
GO:0070098	chemokine-mediated signaling pathway	5.4	1×10^{-2}		
GO:0006720	isoprenoid metabolic process	6.2	1×10^{-2}		
GO:0051281	positive regulation of release of sequestered calcium ion into cytosol	9.5	1×10^{-2}		
GO:0035587	purinergic receptor signaling pathway	9.5	1×10^{-2}		
GO:1990868	response to chemokine	5.1	1×10^{-2}		
GO:1990869	cellular response to chemokine	5.1	1×10^{-2}		
GO:0090026	positive regulation of monocyte chemotaxis	11.5	2×10^{-2}		
GO:0014065	phosphatidylinositol 3-kinase signaling	4.7	2×10^{-2}		
GO:0046942	carboxylic acid transport	3.2	4×10^{-2}		
GO:0035588	G protein-coupled purinergic receptor signaling pathway	9.2	4×10^{-2}		
GO:0015711	organic anion transport	2.7	4×10^{-2}		
GO:0072677	eosinophil migration	13.7	4×10^{-2}		
GO:0031272	regulation of pseudopodium assembly	13.7	4×10^{-2}		
GO:0070167	regulation of biomineral tissue development	4.5	4×10^{-2}		
GO:0015893	drug transport			6.4	4×10^{-3}
GO:1902475	L-alpha-amino acid transmembrane transport			14.6	9×10^{-3}
GO:0015807	L-amino acid transport			13.7	1×10^{-2}
GO:0015813	L-glutamate transmembrane transport			20.9	1×10^{-2}
GO:0006721	terpenoid metabolic process			11.4	2×10^{-2}
GO:1903825	organic acid transmembrane transport			8.0	2×10^{-2}
GO:1905039	carboxylic acid transmembrane transport			8.0	2×10^{-2}

GO:0003333	amino acid transmembrane transport											10.2	2×10^{-2}
GO:0042063	gliogenesis											4.8	2×10^{-2}
GO:0015800	acidic amino acid transport											13.7	3×10^{-2}
KEGG Pathways[2]													
ssc04724	Glutamatergic synapse	5.0	1×10^{-8}	3.4	3×10^{-2}	4.4	3×10^{-4}	3.0	1×10^{-2}			5.3	6×10^{-4}
ssc05033	Nicotine addiction	7.8	9×10^{-8}	13.5	6×10^{-8}	7.8	3×10^{-4}			21.1	3×10^{-5}	11.1	1×10^{-4}
ssc04727	GABAergic synapse	5.1	9×10^{-8}	5.0	3×10^{-3}					12.7	3×10^{-5}	5.6	2×10^{-3}
ssc04713	Circadian entrainment	4.1	2×10^{-5}	4.0	2×10^{-2}	3.2	4×10^{-2}						
ssc04540	Gap junction	4.2	2×10^{-5}	4.0	3×10^{-2}	3.9	7×10^{-3}	4.1	5×10^{-4}				
ssc04971	Gastric acid secretion	4.4	3×10^{-5}			3.6	3×10^{-2}	3.5	1×10^{-2}				
ssc04725	Cholinergic synapse	3.6	3×10^{-5}	3.3	4×10^{-2}	3.3	1×10^{-2}	2.7	2×10^{-2}				
ssc00100	Steroid biosynthesis	8.9	3×10^{-5}					10.6	4×10^{-5}				
ssc05031	Amphetamine addiction	4.6	3×10^{-5}	6.6	5×10^{-4}	5.8	3×10^{-4}					7.4	4×10^{-4}
ssc05032	Morphine addiction	3.8	9×10^{-5}	4.7	4×10^{-3}					11.9	3×10^{-5}		
ssc05030	Cocaine addiction	5.0	2×10^{-4}	8.0	5×10^{-4}	4.7	2×10^{-2}					6.7	1×10^{-2}
ssc04726	Serotonergic synapse	3.1	1×10^{-3}										
ssc03320	PPAR signaling pathway	3.6	2×10^{-3}										
ssc04970	Salivary secretion	3.4	3×10^{-3}			3.5	4×10^{-2}						
ssc04976	Bile secretion	3.4	5×10^{-3}										
ssc00900	Terpenoid backbone biosynthesis	6.1	5×10^{-3}					7.2	4×10^{-3}				
ssc04918	Thyroid hormone synthesis	3.3	6×10^{-3}			4.7	2×10^{-3}	3.9	4×10^{-3}			5.2	1×10^{-2}
ssc04961	Endocrine and other factor-regulated calcium reabsorption	4.0	9×10^{-3}										
ssc04720	Long-term potentiation	3.3	1×10^{-2}			4.0	2×10^{-2}						
ssc04913	Ovarian steroidogenesis	3.6	2×10^{-2}					4.3	1×10^{-2}			5.6	5×10^{-2}
ssc04911	Insulin secretion	2.8	2×10^{-2}			4.5	2×10^{-3}						
ssc04670	Leukocyte transendothelial migration	2.5	2×10^{-2}					2.7	2×10^{-2}				
ssc04916	Melanogenesis	2.6	2×10^{-2}	4.3	8×10^{-3}	3.4	2×10^{-2}						
ssc00590	Arachidonic acid metabolism	3.1	2×10^{-2}					3.2	3×10^{-2}				
ssc04640	Hematopoietic cell lineage	2.7	3×10^{-2}			4.6	7×10^{-4}	4.0	5×10^{-4}				
ssc04925	Aldosterone synthesis and secretion	2.6	3×10^{-2}			3.7	1×10^{-2}					4.0	5×10^{-2}
ssc04666	Fc gamma R-mediated phagocytosis	2.6	4×10^{-2}					3.1	2×10^{-2}				
ssc00910	Nitrogen metabolism	5.3	5×10^{-2}										
ssc04512	ECM-receptor interaction			5.8	5×10^{-4}	5.1	3×10^{-4}						
ssc04974	Protein digestion and absorption			5.4	7×10^{-4}	5.6	1×10^{-4}						
ssc05217	Basal cell carcinoma			5.2	1×10^{-2}								
ssc05412	Arrhythmogenic right ventricular cardiomyopathy (ARVC)			5.0	1×10^{-2}								

ssc03030	DNA replication	6.7	2×10^{-2}								
ssc04110	Cell cycle	3.5	2×10^{-2}								
ssc00534	Glycosaminoglycan biosynthesis	7.8	3×10^{-2}					16.7	4×10^{-2}		
ssc05204	Chemical carcinogenesis	4.6	3×10^{-2}					11.0	5×10^{-3}		
ssc04721	Synaptic vesicle cycle			6.1	3×10^{-4}	3.3	3×10^{-2}			6.0	7×10^{-3}
ssc05146	Amoebiasis			4.8	3×10^{-4}						
ssc05340	Primary immunodeficiency			7.6	8×10^{-4}						
ssc05320	Autoimmune thyroid disease			6.3	8×10^{-4}						
ssc04933	AGE-RAGE signaling pathway in diabetic complications			3.7	6×10^{-3}						
ssc05142	Chagas disease (American trypanosomiasis)			3.6	7×10^{-3}						
ssc04660	T cell receptor signaling pathway			3.3	2×10^{-2}						
ssc05143	African trypanosomiasis			5.6	2×10^{-2}	4.6	2×10^{-2}				
ssc05330	Allograft rejection			4.9	3×10^{-2}						
ssc04612	Antigen processing and presentation			3.8	5×10^{-2}						
ssc04650	Natural killer cell mediated cytotoxicity			3.0	5×10^{-2}						
ssc04610	Complement and coagulation cascades			3.2	5×10^{-2}	3.1	2×10^{-2}				
ssc05150	Staphylococcus aureus infection					7.1	4×10^{-6}				
ssc04611	Platelet activation					3.3	1×10^{-3}				
ssc04064	NF-kappa B signaling pathway					3.3	8×10^{-3}				
ssc04625	C-type lectin receptor signaling pathway					3.0	1×10^{-2}				
ssc04923	Regulation of lipolysis in adipocytes					3.7	2×10^{-2}				
ssc00565	Ether lipid metabolism					4.0	2×10^{-2}				
ssc05133	Pertussis					3.1	3×10^{-2}				
ssc05144	Malaria					3.7	3×10^{-2}				
ssc04380	Osteoclast differentiation					2.4	4×10^{-2}				
ssc01040	Biosynthesis of unsaturated fatty acids					4.5	4×10^{-2}				
ssc00650	Butanoate metabolism									8.8	3×10^{-2}

[1] Gene Ontology.

[2] KEGG = Kyoto Encyclopedia of Genes and Genomes pathway.

[3] NES > 0 (NES < 0) denotes gene over-expression (under-expression) in control relative to MIA-exposed pigs, pigs exposed to weaning stress relative to not stressed (nursed), and males relative to females.

Supplemental Table S3. Log_2(fold change) of the genes in the ensheathment of neurons Kyoto Encyclopedia of Genes and Genomes pathway for the 12 pairwise contrasts between groups characterized by the maternal immune activation, weaning and sex level.

Gene Symbol	A	B	C	D	E	F	G	H	I	J	K	L
Cldn11	0.32	-0.21	0.01	-0.18	0.3	-0.42	0.63	-0.11	-0.25	0.54	-0.28	-0.29
Olig2	0.49	0.25	0.85	-0.59	-0.2	-0.25	-0.03	-0.83	-0.59	-1.31	-0.61	0.23
Mal	1.53	0.96	-0.85	-1.7	-0.39	-0.58	1.11	-1.5	-1.46	2.15	-1.66	0.98
Plp1	1.58	0.51	-0.55	-1.47	0.76	-0.83	0.35	-1.42	-1.74	0.48	-1.62	0.38
Id4	-1.05	-0.66	-0.47	1.11	-1.19	0.17	-1.3	1.12	1.00	-0.26	0.94	-0.8
Ctnnb1	-1.05	-0.81	0.7	1.16	0.07	0.24	0.12	1.12	1.11	-0.97	1.09	-0.85
Mpp5	-0.94	-0.8	0.81	1.11	0.28	0.19	0.02	1.03	1.02	-1.00	1.05	-0.83
Cd9	-0.85	-0.85	0.9	0.93	0.56	0.1	0.53	0.89	1.00	-0.7	1.01	-0.76
Sox10	0.82	0.23	-0.12	-0.96	-0.51	-0.61	0.72	-0.91	-0.76	0.9	-0.94	0.24
Gal3st1	0.37	-0.49	0.6	-0.16	1.28	-0.56	0.3	-0.26	-0.22	0.28	-0.02	-0.34
Jam3	-0.55	-0.7	-0.29	0.63	0.18	-0.09	0.42	0.81	0.69	0.89	0.65	-0.65
Mpz	-1.25	2.73	-2.58	0.57	-2.41	3.11	-2.73	0.78	0.81	-0.04	0.73	2.75
Kcnj10	0.59	-0.17	0.98	-0.45	1.28	-0.47	-0.16	-0.72	-0.6	-0.98	-0.33	-0.05

A = Ma.Nu.Mi vs Ma.Nu.Co; B = Ma.We.Mi vs Ma.We.Co; C = Fe.Nu.Mi vs Fe.Nu.Co; D = Fe.We.Mi vs Fe.We.Co; E = Ma.We.Co vs Ma.Nu.Co; F = Ma.We.Mi vs Ma.Nu.Mi; G = Fe.We.Co vs Fe.Nu.Co; H = Fe.We.Mi vs Fe.Nu.Mi; I = Ma.Nu.Co vs Fe.Nu.Co; J = Ma.Nu.Mi vs Fe.Nu.Mi; K = Ma.We.Co vs Fe.We.Co; L = Ma.We.Mi vs Fe.We.Mi.

Mi = maternal immune activation, Co = control, We = weaned, Nu = nursed, Ma = male, Fe = female.
